# Adaptation of Archaeal Communities to Summer Hypoxia in the Sediment of Bohai Sea

**DOI:** 10.1002/ece3.70768

**Published:** 2025-01-08

**Authors:** Xiaoxiao Guo, Yanying Li, Guisheng Song, Liang Zhao, Jing Wang

**Affiliations:** ^1^ Tianjin Key Laboratory of Animal and Plant Resistance, Tianjin Key Laboratory of Conservation and Utilization of Animal Diversity, College of Life Science Tianjin Normal University Tianjin China; ^2^ School of Marine Science and Technology Tianjin University Tianjin China; ^3^ College of Marine and Environmental Sciences Tianjin University of Science and Technology Tianjin China

**Keywords:** archaea, Bohai Sea, hypoxia, sediment

## Abstract

Understanding the adaptation of archaea to hypoxia is essential for deciphering the functions and mechanisms of microbes when suffering environmental changes. However, the dynamics and responses of archaea to the sedimentary hypoxia in Bohai Sea are still unclear. In this study, the diversity, composition, and distribution of archaeal community in sediment along an inshore–offshore transect across the oxygen‐depleted area in the Bohai Sea were investigated in June, July, and August of 2021 by employing high‐throughput sequencing of 16S rRNA gene. Results indicated that the archaeal communities were dominated by Thermoproteota (80.61%), Asgardarchaeota (8.70%), and Thermoplasmatota (5.27%). Dissolved oxygen (DO) and NO_3_^−^ were the two key factors shaping the distribution of archaeal communities, accounting for 49.5% and 38.3% of the total variabilities (*p* < 0.05), respectively. With the intensity of oxygen depletion, the diversity of archaeal communities increased significantly. Microbial networks revealed that Bathyarchaeia played a key role in interacting with both bacteria and other archaeal groups. Furthermore, adaptions to hypoxia of archaea were also displayed by variation in relative abundance of the predicted ecological functions and the metabolic pathways. The enrichment of specific nitrogen transformation enzymes showed the potential for nitrogen fixation and removal, which might contribute to the balance of N budget and thus facilitate the ecological restoration under eutrophication in Bohai Sea. Our results provided a new picture on ecological and metabolic adaptions to hypoxia by archaea, which will be beneficial to further investigations in extreme environments both theoretically and practically.

## Graphical abstract

The spatial–temporal variation of DO leads to distinct switches in community composition and abundance of archaea. Diversity of archaea communities increased significantly with the intensity of oxygen depletion. Specific nitrogen transformation enzymes encoded by archaea ensure the balance of N budget in hypoxia zone of Bohai Sea.

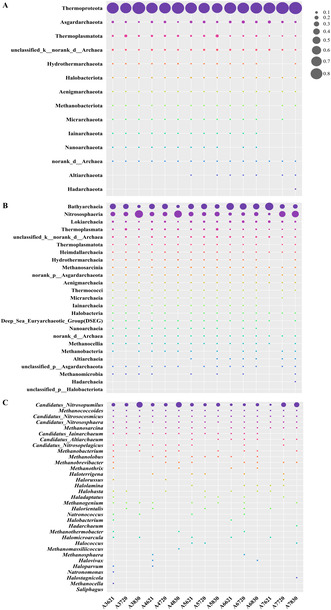

## 1 Introduction

Archaea with chemoorganotrophic/chemolithotrophic metabolic capabilities play vital roles during evolution in life history. They are well known as extremophiles, containing species capable of hyperthermophiles, halophiles, and acidophiles (Cavicchioli 2011; Wang et al. 2017; Baker et al. 2020; Shu and Huang 2022). Among archaea, most halophilic species use amino acids or organic acids as electron donors to obtain vitamins and achieve growth under aerobic conditions (Oren 2015). Additionally, the unique archaea, like methanogens, are mesophilic and nonhalophilic obligate anaerobes, which can convert acetate, CO_2_, and methylated substrates to methane (CH_4_) to make a living (Demirel and Scherer 2008; Costa and Leigh 2014). At the same time, many species of archaea are not extremophiles, with wide distributions in terrestrial (soils) and aquatic (sediments, oceans, and lakes) ecosystems and close associations with animals (Xue et al. 2019; Takai and Nakamura 2011; Oren 2002; Hoshino and Inagaki 2019; Moissl‐Eichinger et al. 2018; Saengkerdsub and Ricke 2014). They participate mostly in biogeochemical cyclings, including carbon, sulfur, phosphorus (Offre, Spang, and Schleper 2013; Evans et al. 2019; Liu, Beer, and Whitman 2012), and nitrogen cycles, such as nitrogen fixation and nitrification (ammonia‐oxidation) (Alves et al. 2018; Berg et al. 2015; Dekas, Poretsky, and Orphan 2009), highlighting the potential significance of archaea in ecological functions.

Hypoxia in marine ecosystems is one of the consequences of anthropogenic pollution and global climate change, which leads to nitrogen release to the atmosphere through denitrification and anaerobic ammonium oxidation (Anammox) (Hutchins and Capone 2022; Fuchsman and Stüeken 2021; Suter et al. 2021; Song et al. 2020). Archaea that participate in carbon and nitrogen cyclings are able to utilize organic/inorganic electron donors and receptors for various metabolic reactions in marine oxygen minimum zones (OMZs), affecting the production and emission of greenhouse gases such as nitrous oxide (N_2_O) and CH_4_ (Stein 2020). Furthermore, the nitrogen fixation mediated by archaea, especially ammonia‐oxidizing archaea (AOA), is also an important portion of marine nitrogen cycle (Berg et al. 2015). In fact, a great number of researches have reported the dominant role of archaea played in ammonia oxidation over bacteria in extreme environments, including hypoxia (Caffrey et al. 2007; Liu et al. 2015; Wang et al. 2017; Pan et al. 2018; Liu, Jing, and Wang 2023). In the OMZs, low oxygen limits the diversity and function of certain aerobic microorganisms and provides special niches beneficial for anaerobic and microaerophilic archaea, such as *Thaumarchaeota*, that could utilize alternative metabolic pathways like anaerobic ammonia oxidation to thrive in low‐oxygen environments (Gu et al. 2022; Kerou et al. 2021). In addition, anthropogenic factors may intensify the eutrophication and consequently fasten the process of hypoxia in coastal and marine environments, leading to shifts in biodiversity and metabolic activities of archaea (Howarth et al. 2011; Li et al. 2020; Lin and Lin 2022). Furthermore, hypoxia also could be exacerbated by stratification in water columns and limitation in oxygen replenishment under global warming, and then facilitate the dominance of anaerobic archaea (Deutsch, Penn, and Lucey 2024). The above changes in turn have cascading effects on marine food webs and overall ecosystem health. Our understanding of how microbial and element cycling processes interact to influence the functional diversity of archaea has expanded significantly in recent years, and studies involving archaea in hypoxic areas such as the Arabian Sea, the Black Sea, the Baltic Sea, the Yangtze River estuary, and the Pearl River estuary have revealed the dominant groups of archaeal communities and the main factors affecting their composition and distribution (Vipindas et al. 2018; Jessen et al. 2017; Berg et al. 2015; Zou et al. 2022; Liu et al. 2014). Other than hypoxic environments, archaea have also been estimated to account for approximately 87% of all prokaryotic cells in deep‐sea sediments with key ecological roles in element cycling (Hoshino and Inagaki 2019). Nonetheless, knowledge about the archaeal response to hypoxia in marine sediment is still limited.

Bohai Sea is located in the northeast of Chinese Mainland, semienclosed with shallow water. Recent depictions of the hypoxic areas in Bohai Sea have focused either on the formation and expansion of OMZs (Zhai et al. 2012), or on the diversity of well‐classified microorganisms (Guo et al. 2022). The intensification of summer oxygen consumption in the Bohai seawater has been ascribed to stratification, acidification, eutrophication, and microbial decomposition of organic substances (Song et al. 2020; Zhang et al. 2022; Wei et al. 2019; Zhao et al. 2017). Our previous study has shown high functional diversity of bacteria harbored in the sediments of the Bohai Sea, and the bacterial community switched distinctly when oxygen depletion occurred (Guo et al. 2022). However, the archaeal compositions, dynamics, and metabolism responses to hypoxia in sediment are still unknown.

In this study, in order to discover the linkage of the biogeochemical variables with archaea in the Bohai sea, a total of 15 sediment samples were collected covering oxygen depletion gradient both spatially and temporally. To better understand how archaea response and adapt to low oxygen conditions, the environmental parameters were collected *in situ*, and nutrients were measured in laboratory; the archaeal community was analyzed by high‐throughput sequencing of 16S rRNA gene. In addition, the mutual responses to environmental variables of archaea and interactions between archaea and bacteria were revealed by networking analysis. Results from this study provide new insightful knowledge on specific ecological adaptions of archaea under hypoxia conditions, and the functional responses of archaeal community to both biological and nonbiological variables in Bohai Sea are detected and discussed.

## 2 Materials and Methods

### 2.1 Sample Collection

**FIGURE 1:**
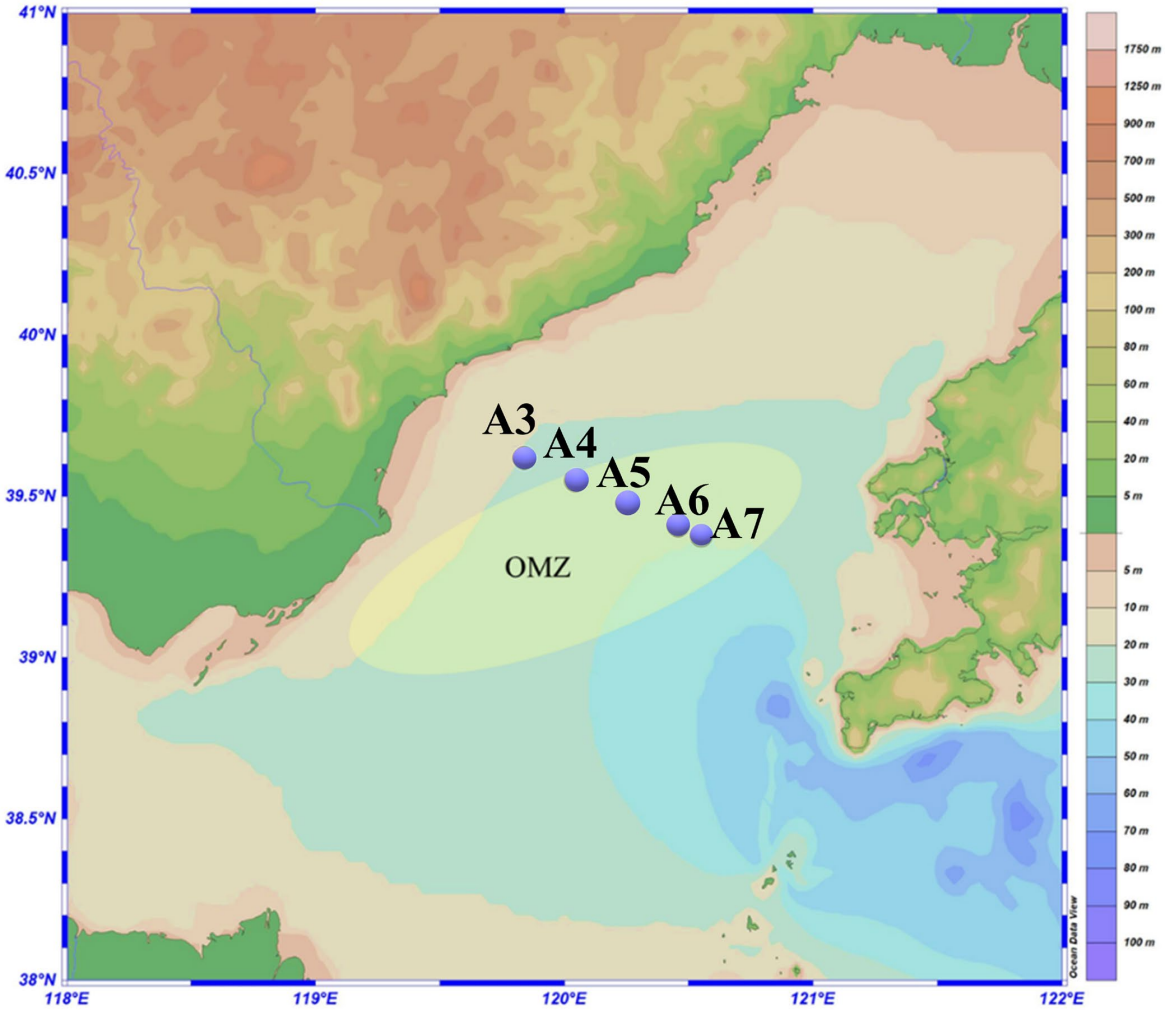
Sampling sites in Bohai Sea along inshore–offshore transect. OMZ (yellow zone) represents the reported hypoxia zone.

Sediment samples were collected from three research cruises conducted in June, July, and August of 2021 in the Bohai Sea. These cruises followed a consistent inshore–offshore transect ranging from Stations A3 to A7 (latitude 39.38° N to 39.69° N, longitude 119.63° E to 120.55° E) (Figure 1, Table S1), which was strategically chosen to cover nonhypoxia (A3) and reported core hypoxia (A4‐A7, Zhai et al. 2012) areas across coastal to offshore regions. In total, 15 sediment samples were collected from five sites in 3 months. A Gray‐O'Hara box corer was employed to minimize the minimal disruption to the sediment structure. Upon retrieval, the top 0–2 cm surface sediment was carefully removed to eliminate any potential disturbances from the overlying seawater, which could affect the integrity of the samples. The collected sediments were then homogenized by thorough mixing to ensure uniformity, and subsamples were randomly taken from the homogenized bulk for downstream analysis. Every 5 g subsamples were collected and sealed in sterile, airtight bags immediately. For DNA extraction and high‐throughput sequencing, the samples were transported to the laboratory on dry ice and stored at −80°C until further processing to preserve the integrity of nucleic acids; for chemical analysis, the samples were transported to the laboratory on ice and treated immediately after arrival.

### 2.2 Environmental Parameter Measurement

Essential parameters, including salinity, dissolved oxygen (DO), pH, and temperature, were measured *in situ* from seawater by using a SeaBird SBE9 conductivity–temperature–depth (CTD) recorder (Sea‐Bird Electronics) at each sampling site. Nutrient concentrations, including ammonium (NH_4_^+^), nitrate (NO_3_^−^), and nitrite (NO_2_^−^), were determined using an AA3 nutrient Auto Analyzer (SEAL Analytical, United States) from sediment pore water. In brief, 5 g sediment for each sample was centrifuged at 5000 rpm for 30 min (Liu et al. 2023). The analyses were performed with two identical channels of the AA3 for simultaneous analyses of NO_3_^−^/NO_2_^−^ and NH_4_^+^, according to the same standard operating procedures (SOP). The analytical protocols used for the autoanalyzers were based on Technicon methodologies for NO_3_^−^/NO_2_^−^ and NH_4_^+^, with the conditions specified by the method for the AA3 (Technicon 1973). All measurements were performed at a rate of 30 samples per hour and a 4:1 sample‐to‐wash ratio (by volume).

### 2.3 DNA Extraction and High‐Throughput Sequencing

The DNeasy Power Soil Kit (QIAGEN) was used to extract total DNA from 0.5 g sediment samples, and the specific steps were followed according to the manufacturer's instructions. Total DNA of sediment samples was amplified in the 9700 PCR thermal cycler (ABI GeneAmp) and following the published procedures (Guo et al. 2022) with replacement of primer sets targeting the V4 and V5 regions of archaeal 16S rRNA gene. Phylogenetically diagnostic sequences were amplified using the universal primers 524F10extF (5′‐TGYCAGCCGCCGCGGTAA‐3′) and Arch958RmodR (5′‐YCCGGCGTTGAVTCCAATT‐3′) (Ma et al. 2020). The final PCR product was loaded onto 2% agarose gel for electrophoresis to monitor the accuracy of amplified fragments. High‐throughput sequencing was performed using Illumina MiSeq system (Illumina MiSeq, United States).

### 2.4 Statistical Analysis

Silva138.2 (released in July 2024) was used to annotate the sequences. This version of the SILVA database marked most updated archaeal taxonomy, incorporating the reclassification proposed by recent phylogenetic studies. Nonrepetitive sequences were extracted from optimized sequences in Usearch (v.7.0, http://drive5.com/uparse/), which were clustered into operational taxonomic units (OTUs) based on 97% similarity (Edgar 2010). Mothur (1.30) was used to analyze the high‐throughput sequencing data and calculate the diversity indices under random sampling. Alpha diversity indices (Shannon, Simpson, ACE, and Chao 1) were then calculated and nonmetric multidimensional scaling analysis (NMDS) based on binary Jaccard distance for beta diversity was calculated between the five sites from three sampling times. All data were visualized in R (3.6.3). Archaeal community dynamics at phylum level with eight environmental variables (temperature, salinity, depth, pH, DO, NO_3_^−^, NO_2_^−^, and NH_4_^+^) were analyzed using redundancy analysis using “rdacca.hp” package and mapped with “ggplot2” package in R (Lai et al. 2022). Functional potential of archaeal communities in sediment samples was predicted using PICRUSt (phylogenetic investigation of communities by reconstruction of unobserved states) (Langille et al. 2013). Network analysis was utilized to reveal the interactions among archaeal community, as well as archaea and bacteria in sediment; and Gephi (0.9.2) software was used to visualize networks and perform modular analysis. In addition, significance test was conducted using one‐way ANOVA, and Spearman correlation analysis was performed by employing SPSS (17.0).

### 2.5 Nucleotide Sequences Accession Numbers

The annotated nucleotide sequences of 16S rRNA gene were deposited to the National Center for Biotechnology Information (NCBI) Sequence Read Archive (SRA) database (accession no.: PRJNA1100295).

## 3 Results

### 3.1 Diversity and Distribution of Sedimentary Archaea

**FIGURE 2:**
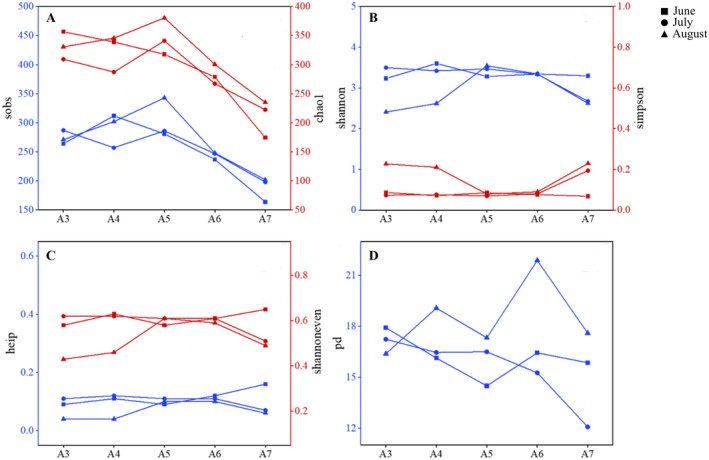
Alpha diversity of 16S rRNA gene‐encoded archaea at each sampling site and time.

In total, 718,238 sequences and 8958 OTUs were generated from 15 samples by high‐throughput sequencing (Figure S1, Table S1). Genes with a similarity higher than 97% compared to Silva (138.2) database were selected for subsequent analysis. The Sobs and Chao indices of the samples showed that the highest richness of archaea species was observed at Site A5 in August, while the lowest richness occurred at Site A7 in June. Changes in the Shannon index varied from inshore to offshore with different sampling times. In June and July, the index decreased first and then increased mostly; however, the index showed an upward and then downward trend in August when hypoxia was more pronounced (Figure 2). From a single‐site perspective, except for Sites A5 and A6, the lowest index always emerged in August. In general, the Shannon even and Heip indices in August were significantly lower than those in June (*p* < 0.05), however, no significance was detected among sampling sites in different months. Regarding the tolerance of archaeal communities to external invasive species, the phylogenetic diversity (PD) analysis was conducted. The results showed that the PD value of offshore sites (A4–A7) in August was significantly higher than that in June and July, indicating that when hypoxia emerged, the tolerance of archaeal communities to the surrounding environment increased significantly. Overall, with the occurrence of hypoxia, the diversity of archaea in August was generally higher than that in June and July, and the fluctuation range of Alpha diversity indices in August samples was significantly greater than that from the other 2 months (Figure 2).

**FIGURE 3:**
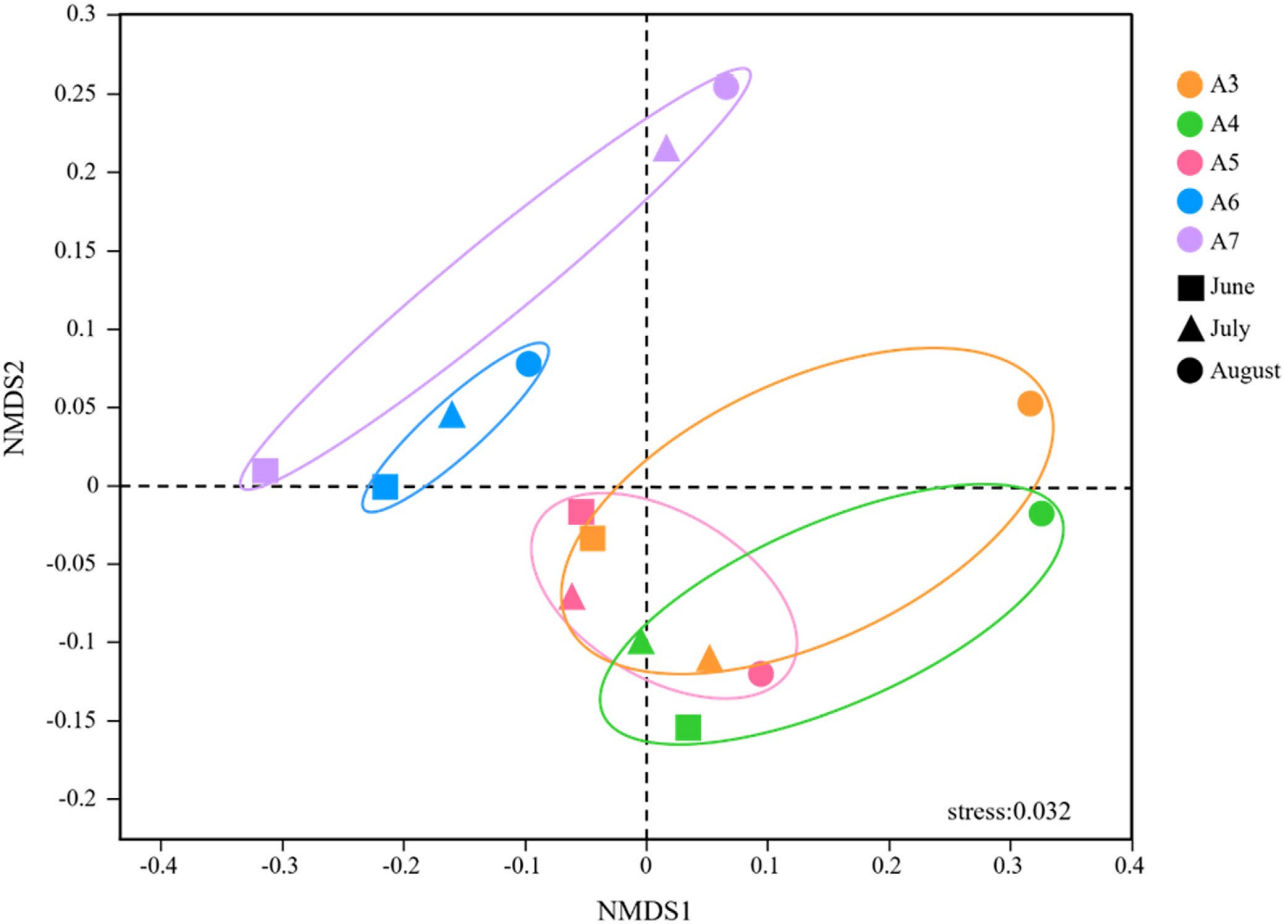
Nonmetric multidimensional scaling (NMDS) plot depicting archaea composition of each sample from different sampling times and sites at phylum level. (Axis defines 2D space that allows the best spatial representation of sample distance based on binary Jaccard distance with stress = 0.032. The points in different colors represent sampling sites and different symbols represent sampling time.)

NMDS analysis for the beta diversity of archaea was conducted based on the binary Jaccard to unveil spatial–temporal distribution (stress = 0.032) (Figure 3). The results showed that samples from different sites gathered together along the inshore–offshore gradient and sequestrated according to site locations. The overlap of samples for A3, A4, and A5 indicates a high similarity in the composition of archaeal communities at these three inshore sites. Correspondingly, samples of A6 and A7 from the offshore were clustered together without overlapping, indicating a high heterogeneity of archaea at these two offshore sites. From a temporal perspective, the samples from June and July were closer and had higher similarity, while those from August were farther away from other two sampling times, which suggests that in August, significant hypoxia resulted in distinct changes in archaea in composition and higher heterogeneity of community between the sampling times. A cluster analysis was further conducted to show the spatiotemporal heterogeneity and similarity of archaeal communities, and the results (Figure S2) indicated that archaea in nearshore stations clustered more closely based on time, while samples in offshore stations clustered mainly based on locations.

### 3.2 Community Composition of Archaea

**FIGURE 4:**
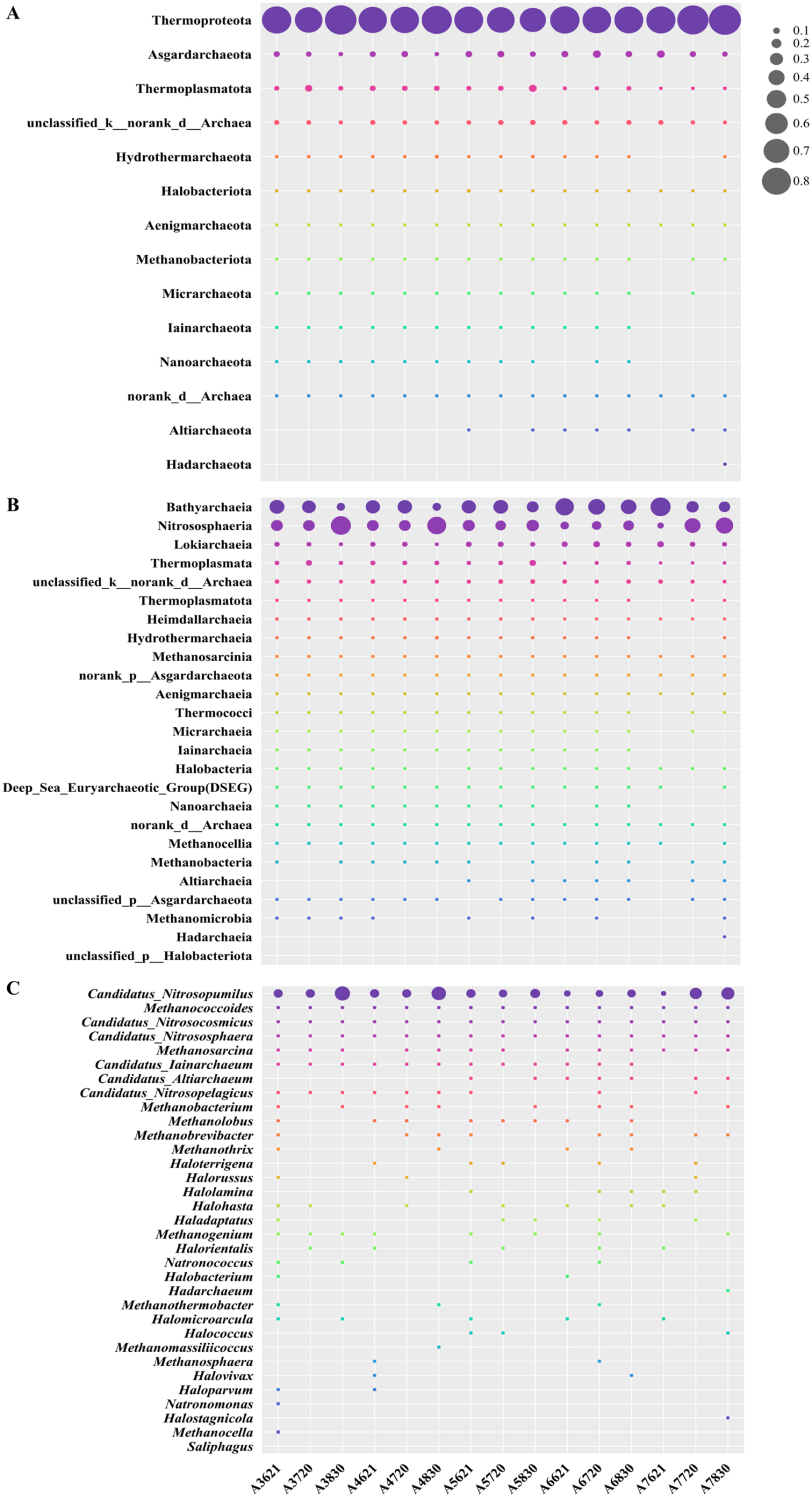
The community composition of dominant archaea at phylum (A), class (B), and genus (C) level from each sampling time and site. (The size of the bubbles indicates the relative abundance of archaea.)

A total of 12 phyla of archaea were identified (Figure 4), among which Thermoproteota (80.61%), Asgardarchaeota (8.70%), and Thermoplasmatota (5.27%) were the Top 3 phyla. At the lowest DO sample (Site A5 in August), the relative abundance of the dominant group Thermoproteota decreased, including the subordinate classes Bathyarchaeia (43.13%) and Nitrososphaeria (37.48%). Their relative abundances showed an opposite trend along sampling time scenario (June–August). Similarly, the second‐dominated phylum Asgardarchaeota also decreased in relative abundance during the occurrence of hypoxia in August. This phylum mainly identified classes: Lokiarchaeia (8.13%) and Heimdallarchaeia (0.40%). Among these, Lokiarchaeia dominated with a relative abundance of 8.13% singularly in all classes affiliated with Asgardarchaeota. And the differences in abundance of Lokiarchaeia were more significant based on sampling time than sampling sties. In addition, the relative abundance of Heimdallarchaeia in offshore sites was significantly higher than that in nearshore sites (*p* < 0.05). It is worth noting that, unlike the above two phyla, the relative abundance of Thermoplasmatota reached its maximum of 13.13% during oxygen depletion, and the relative abundance of offshore sites was significantly lower than that of nearshore sites (*p* < 0.05). Thermoplasmata (4.83%)‐affiliated Thermoplasmatota was the fourth dominant group annotated, and the abundance of this group was higher in the nearshore stations than in the offshore stations. *Candidatus_ Nitrosopumilus* represents the most dominant genus‐level group observed. While its abundance is higher at nearshore sites compared to offshore locations, this difference is not statistically significant. However, during August, a period characterized by elevated DO consumption, the abundance of *Candidatus_Nitrosopumilus* increases significantly (*p* < 0.05). In addition to dominant groups, some rare groups with relatively low abundance (< 0.38%) also exhibited spatial–temporal variation in their distributions. From the subhypoxic core area (A5) extending to sites further offshore (A6 and A7), the abundances of Halobacteriota, Aenigmarchaeota, and Methanobacteriota declined significantly over time, coinciding with the progressive onset of seasonal hypoxia. The distributions of Methanobacteriota, Micrarchaeota, Lainarchaeota, Nanoarchaeota, and Altiarchaeota varied significantly across sampling sites. Altiarchaeota exhibited a notably higher abundance in offshore regions compared to nearshore areas, whereas the other groups showed an opposite pattern, demonstrating a stronger preference for nearshore habitats. At class level, Altiarchaeota (0.16%) and Methanomicrobia (0.03%) showed differences in abundance among sampling locations. Altiarchaeota tended to inhabit offshore sites, while Methanomicrobia, on the contrary, was more abundant in nearshore sites.

### 3.3 Environmental Drivers on Archaea

**FIGURE 5:**
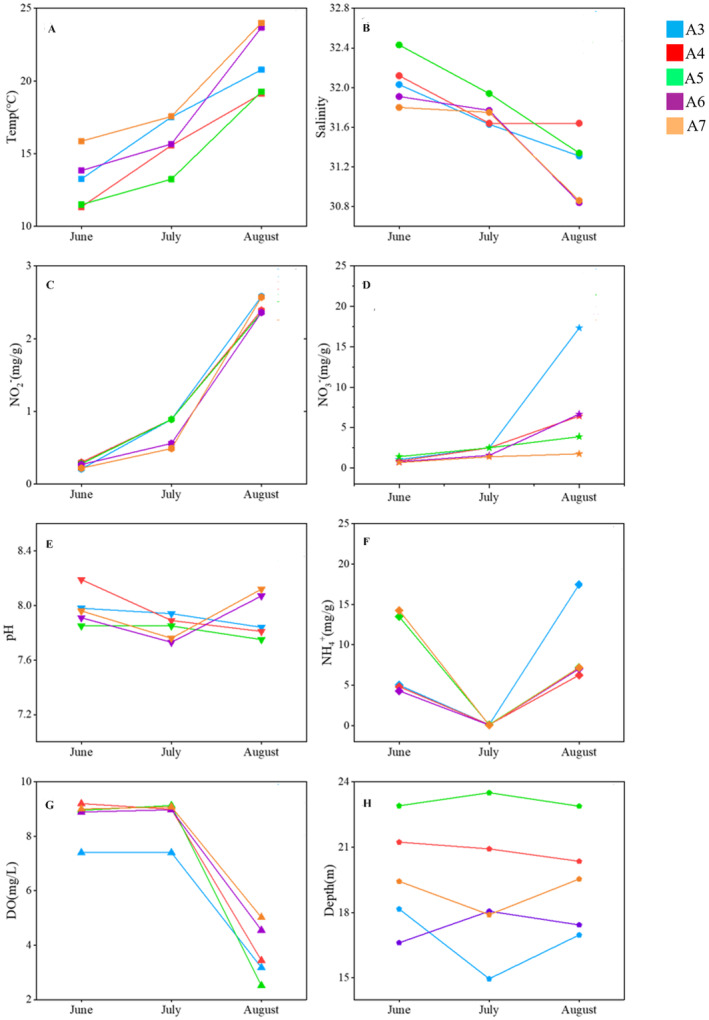
Physiochemical parameters (A: temperature, B: salinity, C: NO_2_^−^, D: NO_3_^−^, E: pH, F: NH_4_^+^, G: DO, and H: depth) of each sediment sample.

Significant variations in temperature, salinity, DO, NO_3_^−^, NO_2_^−^, and NH_4_^+^ contents among sampling time scenarios (*p* < 0.05) were detected, but the differences were not significant among different sampling sites (*p* > 0.05). From June to August, the temperature in the study area increased steadily, while the salinity decreased gradually. There was no significant difference in pH values between either sites or sampling time (*p* > 0.05). The DO value decreased sharply in August, indicating the occurrence of hypoxia from A3 to A7, with Site A5 was the core area where the DO value was the lowest (2.52 mg/L). The contents of NO_3_^−^ and NO_2_^−^ significantly increased in August, and NO_3_^−^ was more enriched than NO_2_^−^ at all sites. Moreover, the content of NH_4_^+^ in August was significantly higher than that in June and July (*p* < 0.05). In summary, there were significant differences in the physical and chemical properties of the study area in August when hypoxia was taking place (Figure 5).

**FIGURE 6:**
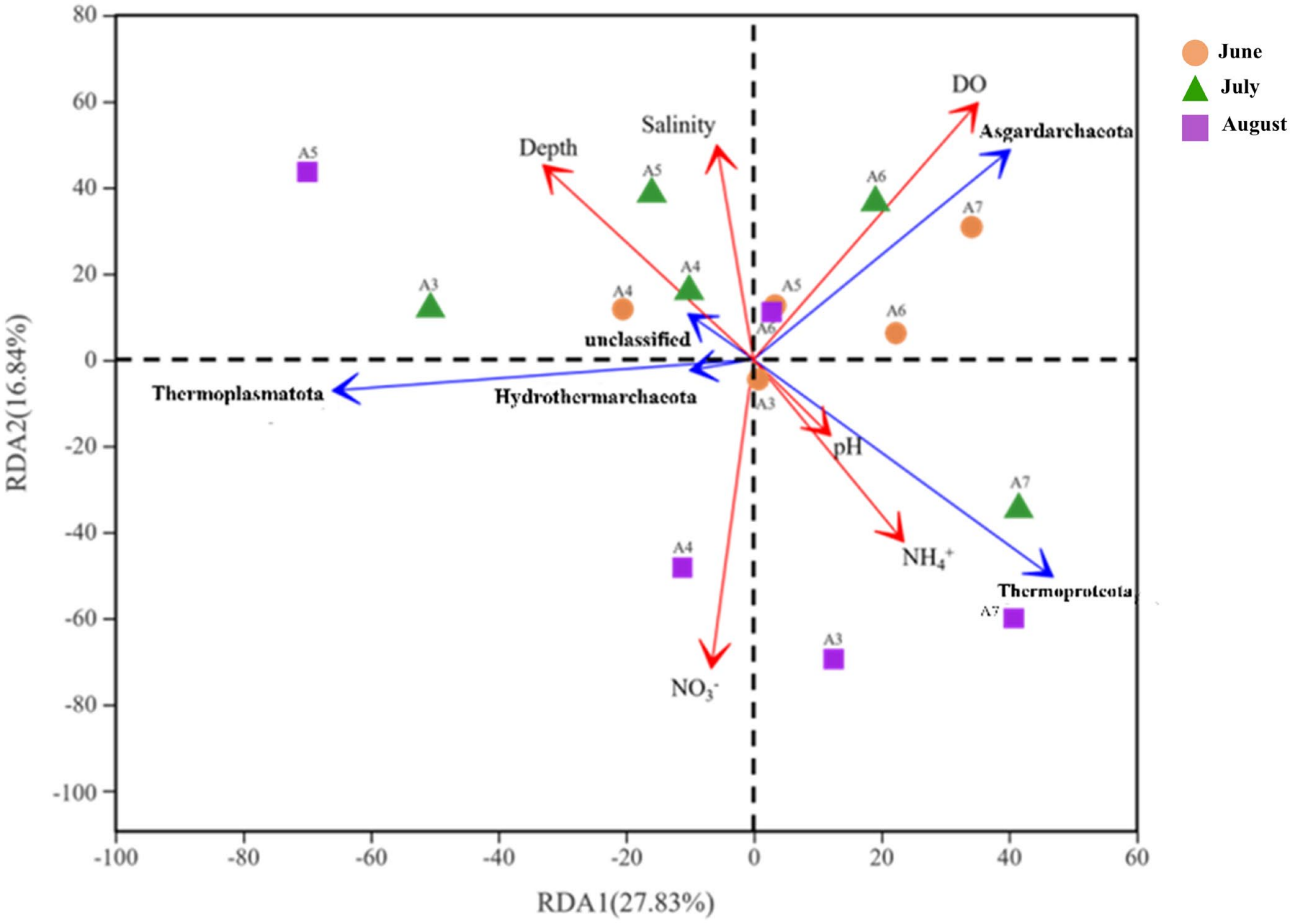
RDA (redundancy analysis) of the archaea distribution and physicochemical parameters. The correlation between environmental variables and the RDA coordinate axis is represented by the length and angle of the red arrow. The blue arrow indicates the Top 5 archaea with relative abundance.

The environmental factors were screened using the variance inflation factor (VIF), then depth, salinity, DO, NO_3_^−^, NH_4_^+^, and pH were selected for RDA analysis since their VIF < 10 (Table S2). The first two axes in the RDA analysis explained 27.83% and 16.84% of the total variations of environmental factors, respectively. The RDA analysis indicated that DO and NO_3_^−^ were the two most important contributing factors to the distribution of archaeal communities, accounting for 49.5% and 38.3% of the total variances (*p* < 0.05), respectively (Figure 6). Almost all samples in July were related to water depth, salinity, and DO, whereas NH_4_^+^ and NO_3_^−^ concentrations were the most influential factors in almost all August samples.

**FIGURE 7:**
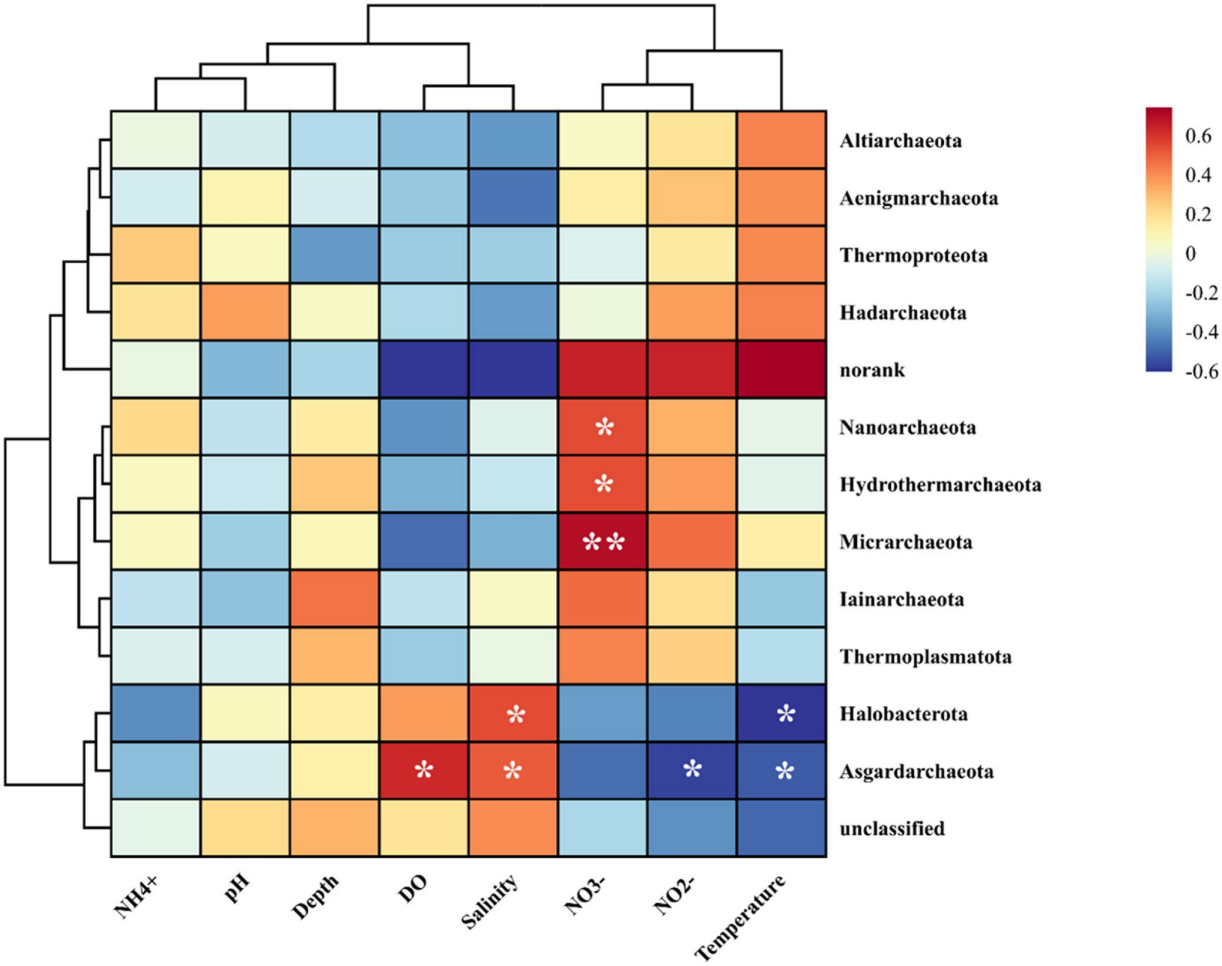
Spearman correlation analysis between physicochemical parameters and archaea at phylum level (* and ** represent significant levels of 0.05 and 0.01, respectively).

Spearman correlation analysis further revealed the relationship between archaeal diversity and environmental parameters (Figure 7). In terms of archaea from different phyla, Asgardarchaeota was closely associated with DO, while Thermoproteota was mainly affected by the concentration of NH_4_^+^ and temperature. Hydrothermarchaeota, Nanoarchaeota, and Micrarchaeia were significantly positively correlated with nitrate (*p* < 0.05). Asgardarchaeota and Halobacteria were positively associated with salinity, while both of them were negatively affected by temperature in significance. Furthermore, linear regression analysis between DO and diversity indices was conducted. As DO decreased, the abundance of Chao, Sobs, and Simpson indices increased, indicating the hypoxic environments strengthened the instability of archaeal communities (Figure S3). The variation of archaeal beta‐diversity index was identical, which showed an upward trend along decreasing DO (Figure S4), suggesting the amplified divergence between archaeal communities with enhancement of hypoxia.

### 3.4 Network Analysis Among Microbiota in Sediments

**FIGURE 8:**
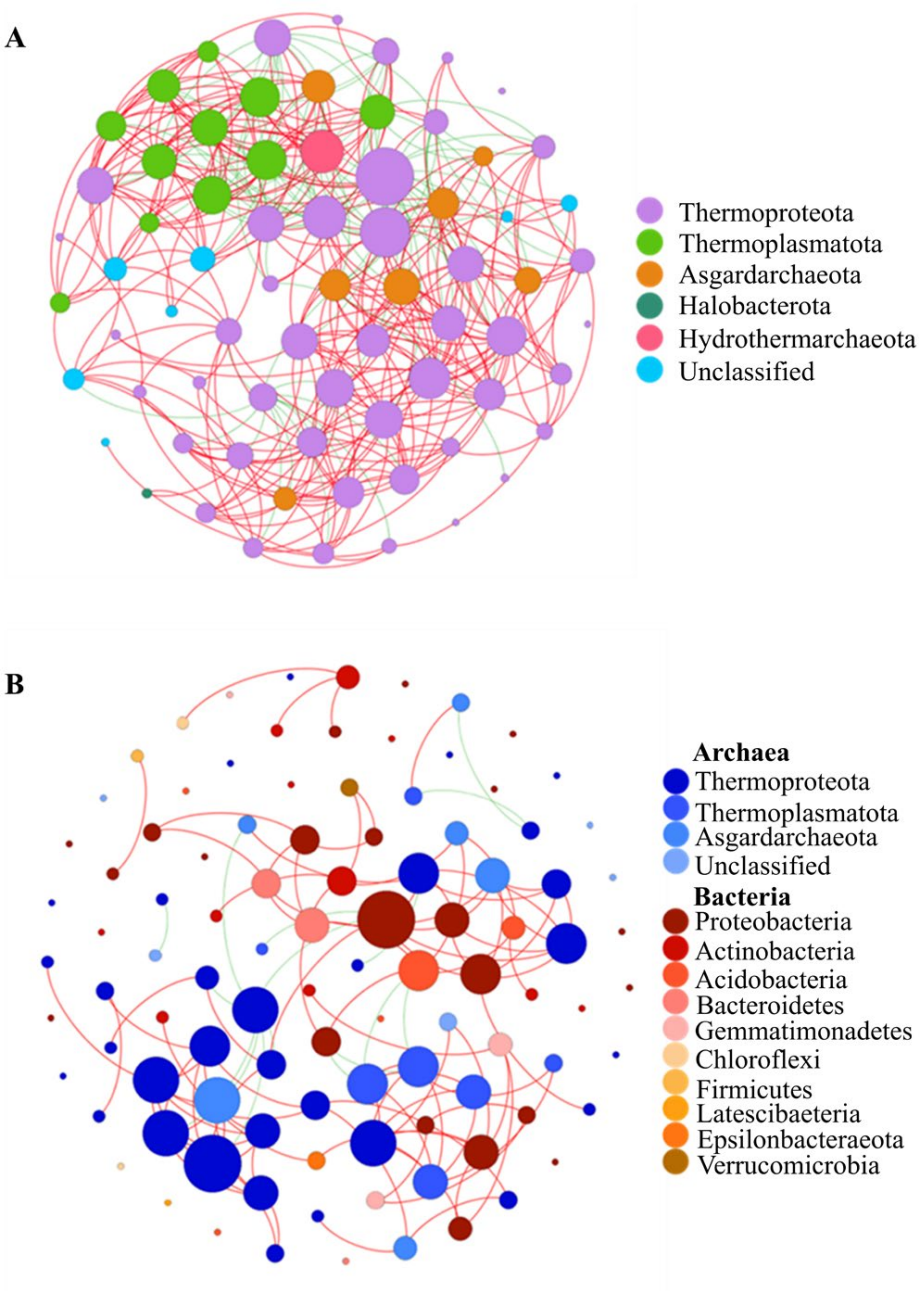
Network analysis of interactions among archaea (A) and archaea versus bacteria (B) based on Spearman correlation. The size of a node is directly proportional to the number of connections, with red and green edges indicating positive and negative correlations, respectively.

Based on Spearman correlation coefficient, major archaeal OTUs with relative abundance higher than 0.1% were selected for network analysis. The results of network comprised 71 nodes and 415 edges, among which 322 edges represented positive correlation and 93 edges represented negative correlation. Clearly, Thermoproteota and Thermoplasmatota were widely distributed, accounting for 77.46% of all nodes (Figure 8A). The Nitrosophaeria of Thermoproteota had a maximum of 32 connections. The highest values of betweenness centrality and eigenvector centrality both appeared in the Bathyarchaeia from Thermoproteota, which revealed the complicated species interconnections with other groups.

In order to reveal the correlation between the diversity and dynamics of archaeal and bacterial communities, network analysis was conducted by the published bacterial community (sequencing of 16S rRNA gene, V3–V4 region; NCBI accession numbers SRR17253936‐ SRR17253955.Guo et al. 2022) data together with current archaeal data from exactly the same sample. The co‐occurrence pattern between archaea and bacteria generated a total of 117 edges, with 111 positive correlations and 16 negative correlations only (Figure 8B). The bacterial community mostly interacted with archaea in the sediment of the Bohai Sea were Bathyarchaeia (OTU4, eigenvector centrality = 0.91) and Deltaproteobacteria (OUT79, eigenvector centrality = 0.29), which contained 18 edges. A positive correlation was also detected between Asgardarchaeota of archaea and Acidobacterium‐ and Proteobacterium‐affiliated bacteria. These findings are beneficial for further exploring the specific mechanisms between these microbial groups.

### 3.5 Function Prediction of Archaea Community

**FIGURE 9:**
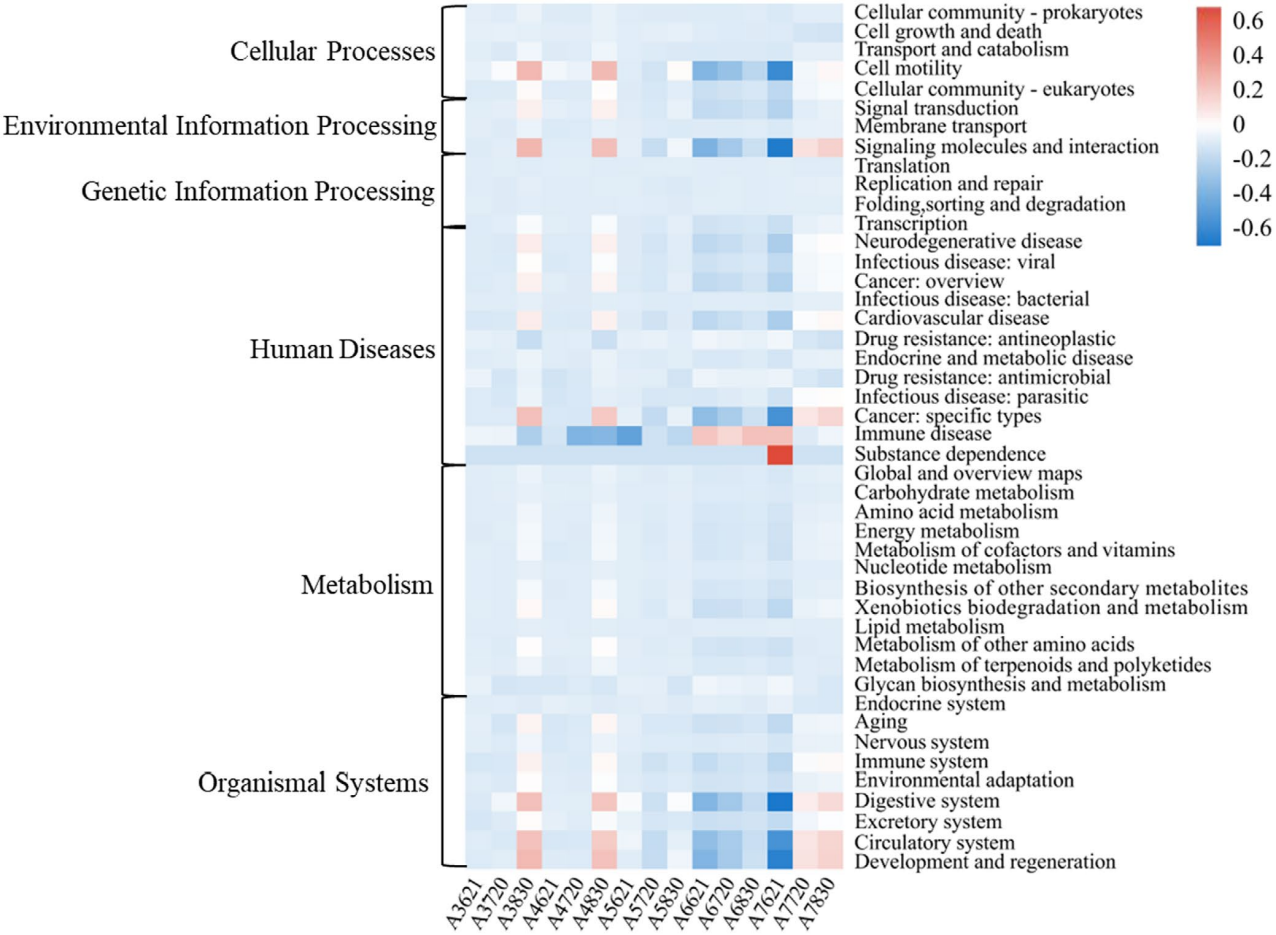
Heatmap of relative abundance of archaea functional categories (Level 2) based on KEEG database.

PICRUSt 2 was performed to predict the functional variation of archaea under DO depletion. In total, six types of biological metabolic pathways were identified using the KEGG (Kyoto Encyclopedia of Genes and Genomes) database, including metabolism, genetic information processing, environmental information processing, cellular processes, human diseases, and organic systems (Figure 9). Among them, the main components were metabolic and genetic information processing, accounting for 77.56% and 12.46% of the total metabolic pathways, respectively. The results identified 45 subfunctions at the secondary functional level, revealing the main functions of archaea include global and overview maps (41.95%), carbohydrate metabolism (8.77%), amino acid metabolism (8.20%), translation (6.64%), energy metabolism (5.43%), metabolism of cofactors and vitamins (4.74%), and nucleotide metabolism (2.95%). It is worth noting that multiple metabolic pathways responded to hypoxia, especially cell motility, signal transduction, signaling molecules and interactions, specific types of cancer, digestive system, and circulatory system. Metabolic pathways such as development and regeneration have increased significantly.

**FIGURE 10:**
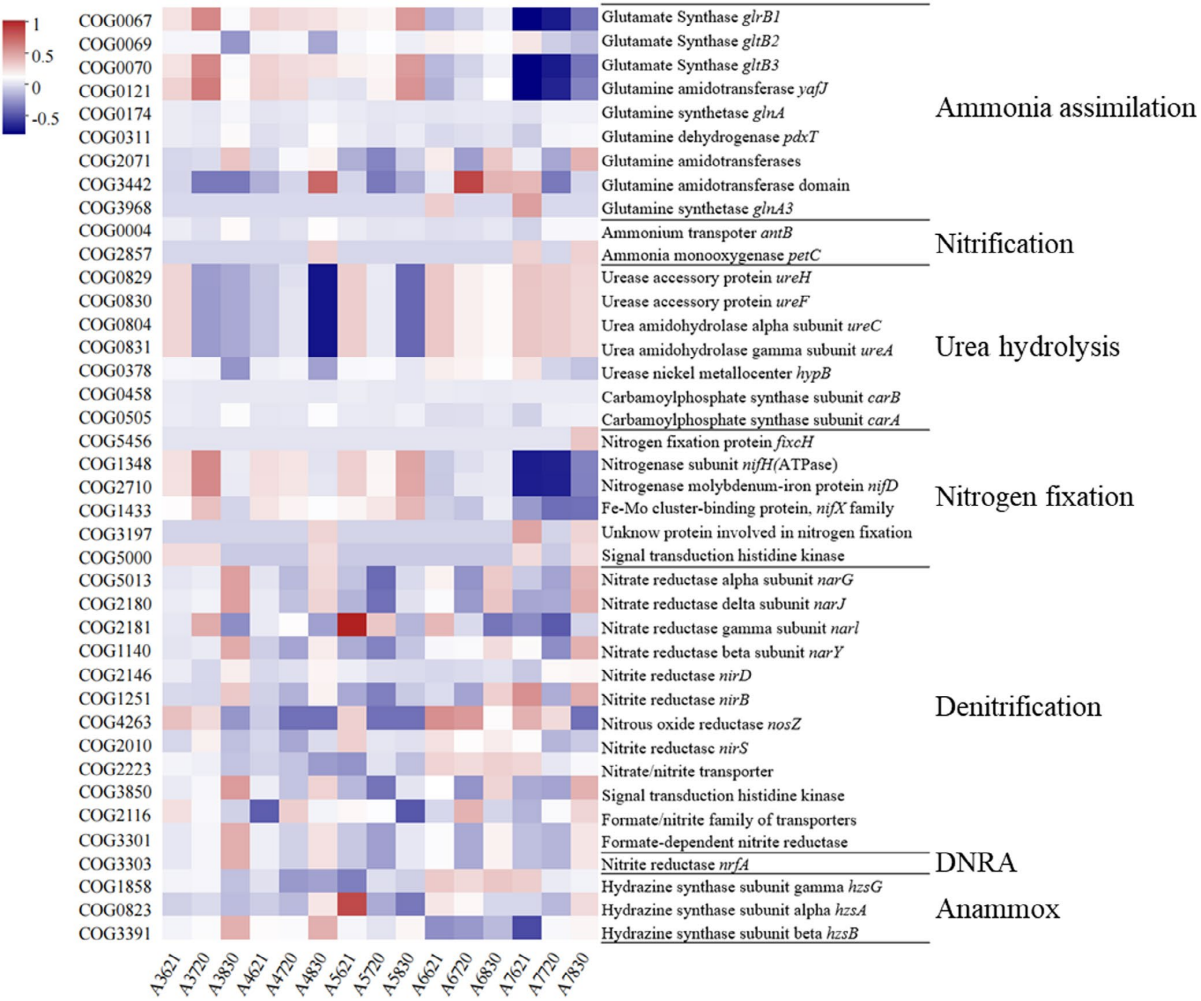
Heatmap of relative abundance of different enzymes and their corresponding genes related to nitrogen cycling encoded by archaea based on COG database.

With a special focus on the relevant genes involved in nitrogen cycling, PICRUSt 2 was performed based on the COG (clusters of pathologies groups) database (Figure 10). Spatially, nitrification, ammonia oxidation, dissimilatory nitrate reduction to ammonium (DNRA), and denitrification showed a trend of first decreasing and then increasing along the gradient from nearshore to offshore. Correspondingly, functional genes related to urea hydrolysis showed a downward trend during the occurrence of hypoxia, while ammonia assimilation, DNRA, nitrification, and ammonia oxidation were enhanced. Meanwhile, the trend of nitrogen fixation was evident in July, with a continuous increase at Site A5 and a decrease at other sites.

**FIGURE 11:**
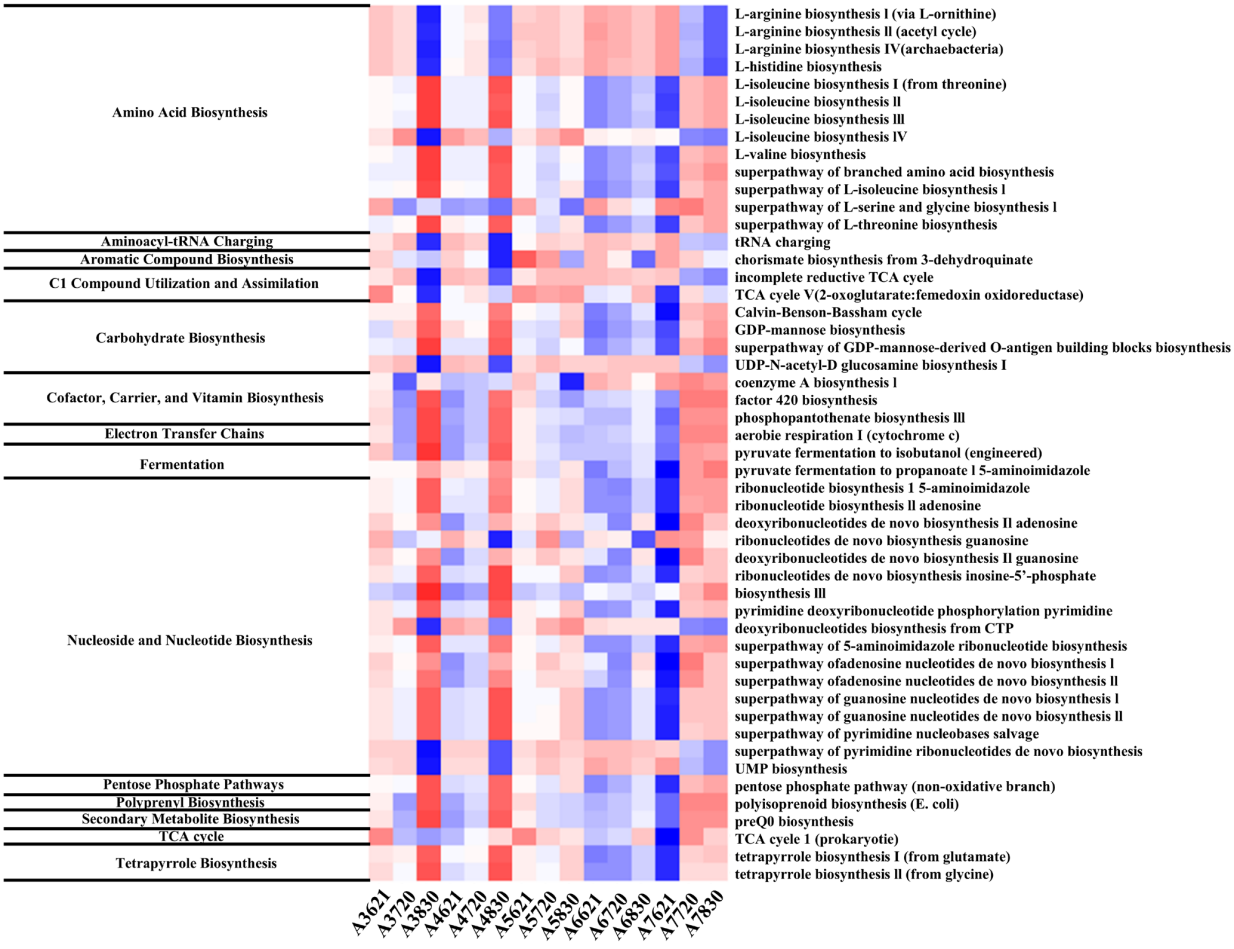
Heatmap of relative abundance of archaea functional categories (Level 2) based on MetaCyc database.

Furthermore, metabolic pathways and enzymes from various fields of life were revealed based on the MetaCycle database. A total of 333 functional pathways were predicted. The results showed the classification and quantitative changes in the Top 50 functional pathways in the sediment of the Bohai Sea, which were divided into 14 different metabolic categories. The total percentage of these pathways from June to August ranged from 79.26% to 81.72%, including amino acid biosynthesis, carbohydrate biosynthesis, cofactor, carrier, and vitamin biosynthesis, nucleoside and nucleotide biosynthesis, TCA cycle, tetrapyrrole biosynthesis, and several other pathways (Figure 11). The main functional pathway of archaea in Bohai sediment displayed distinct variations when hypoxia occurs, with the most representative metabolic pathways including aerobic respiration I (cytochrome c), L‐isoleucine biosynthesis II (L‐isoleucine biosynthesis II), incomplete reduced TCA cycle, superpathway of branched amino acid biosynthesis, and L‐isoleucine biosynthesis IV.

## 4 Discussion

### 4.1 Hypoxia Condition at Bohai Sea in Summer

The strict hypoxia conditions are under debating with the intensive study on oxygen depletion in world oceans. Ever since the define of hypoxia, which was first raised by Robert J. Diaz and Rutger Rosenberg with the DO level < 2 mg/L (Diaz and Rosenberg 1995), a broader range of investigations has been applied worldwide. Vaquer‐Sunyer and Duarte (2008) provide compelling evidence that the conventional hypoxia threshold of 2.0 mg/L may inadequately protect marine biodiversity. Their analysis of species‐specific oxygen sensitivity reveals that negative impacts, including biodiversity loss and ecosystem disruption, can occur at DO levels well above 2.0 mg/L. This observation is consistent with other studies, which insist that hypoxia thresholds may vary widely due to species‐specific tolerances and environmental conditions, with commonly applied thresholds ranging from 2.0 mg/L to 4.0 mg/L (Tellier et al. 2022; Low et al. 2021). Further research demonstrates that sublethal effects on fish, such as reduced food intake and growth, begin significantly at around 4.5 mg/L of DO, a level well above traditional hypoxia thresholds (Hrycik, Almeida, and Höök 2017). Collectively, these findings underscore the need to establish a revised, higher hypoxia threshold that captures both lethal and sublethal impacts, offering a more ecologically relevant benchmark for marine conservation, especially the survival of benthic organisms. A recent study indicates that many marine organisms experience significant impacts when DO levels fall to 3.0 – 4.0 mg/L (Guo et al. 2024). According to the national water quality standard (GB 3097‐1997), DO levels below 4.0 mg/L are classified as severe pollution across four seawater categories. Consequently, the hypoxia threshold in the central Bohai Sea has been set at a DO level of < 4.0 mg/L (Guo et al. 2024).

Ever since the discovery of periodically occurring hypoxia in the Bohai Sea (Zhai et al. 2012), observations on DO showed significant decreases in summer and recover in autumn, displaying an annual cyclic pattern in the studied area (Fennel and Testa 2018; Guo et al. 2022; Liu et al. 2019; Zhao et al. 2021). The lower DO concentration in Bohai Sea is primarily due to limited water exchange (stratification), lower biological productivity, and organic matter degradation (Wu et al. 2024). Nearshore areas suffered from dynamic water exchange due to tides and river inputs, supporting higher primary productivity that increased DO through photosynthesis (Geng et al. 2021; Li et al. 2023), like A3 in this study. In contrast, offshore areas experience weaker currents, fewer nutrients, and stronger thermoclines, which limit oxygen replenishment (Zhao et al. 2017). Additionally, the degradation of organic matter consumes significant oxygen, especially in deeper offshore regions, further reducing DO levels (Zhao et al. 2017). Remarkably, all five sites in August have exhibited hypoxia in this study rather than only site A5, which has been reported as a core hypoxic zone in previous studies and cruises (Guo et al. 2022; Wang et al. 2022), indicating a further expansion of the hypoxic over this area with time preceding. Moreover, the intensify of DO depletion also could be confirmed by concentrations measured from the same site across years (2.52 mg/L in this study and 4.21 mg/L in August 2018) (Guo et al. 2022). Marine oxygen consumption is mainly controlled by both physical and biogeochemical processes (Breitburg et al. 2018; Rabalais et al. 2014). The Bohai Sea has faced a significant transition from a nitrogen‐limited oligotrophic environment to a phosphorus‐limited eutrophic situation, the increased primary productivity of seawater promotes the respiration of microorganisms in sediment, which further consume a large amount of DO, contributing to the intense hypoxic degree (Robinson 2019).

### 4.2 The Archaeal Community in Response to Oxygen Depletion

Thermoproteota has been found to be dominant in sediments from the Peruvian margin, the Cascadian margin, and the Okhotsk Sea, which is consistent with our study (accounting for over 80.68%) (Inagaki et al. 2006, 2003). With the significant occurrence of low oxygen, the relative abundance of Thermoproteota is disturbed, and two descending classes of Thermoproteota, Bathyarchaeia, and Nitrososphaeria also show dynamic response correspondingly. Nitrososphaeria is abundant in low DO samples, whereas Bathyarchaeia displays an opposite trend (Figure 4). Most 16S rRNAs affiliated with Nitrososphaeria have been identified as *Candidatus* (*Ca*.) *Nitrosopumilus*, *Ca. Nitrocosmicus*, and *Ca. Nitrososphaera*, some rare groups are *Ca. Nitrosopelagicus* and tend to inhabit nearshore areas in this study. All of these archaea are involved in nitrogen transformation process as nitrifiers in anoxic marine (Vuillemin 2023). In terms of Bathyarchaeia, *Miscellaneous Crenarchaeota* Group (MCG) is the most abundant and active uncultured archaea in anoxic marine, including Pearl Estuary, Changjiang River Estuary, and northern Gulf of Mexico (Liu et al. 2014; Zou et al. 2022; Devereux et al. 2015). Thus, both Bathyarchaeia and Nitrososphaeria should be induced to hypoxic environments, while their abundance showed an opposite trend under a decreasing trend of oxygen, indicating a certain degree of competition among dominant archaea. Moreover, *Desulfurococcales*, as a rare group, may also play a role in influencing the distribution of Bathyarchaeia, which employ sulfur as an electron acceptor and inorganic nutrient electron source in anaerobic respiration and prefer growing in sulfur‐containing geothermal water or soil, such as sulfur‐rich hot springs in Yellowstone National Park (Jay et al. 2016).

Asgardarchaeota, which is secondly dominated by archaeal community, are considered to be closely linked with eukaryotic origin, exhibit high diversity with an anaerobic lifestyle, and are widely distributed in marine sediments (Bulzu et al. 2019; Salcher et al. 2020; Seitz et al. 2019; Spang et al. 2015). One representative of the Asgard, Lokiarchaeia, which anaerobic utilizes CO_2_ and extracellular polymeric substances of diatoms, has a higher utilization efficiency than the bacteria on average in hypoxic marine sediment (Orsi et al. 2020). Another member of Asgard, Heimdallarchaeia, is the closest acidophilic archaeal lineage to eukaryotes, which may have a microaerophilic niche, engaging in unique aerobic metabolic pathways such as the Kynurenine pathway (Bulzu et al. 2019). Therefore, the decrease in Asgardarchaeota at low oxygen sites may be due to the dependence on oxygen for aerobic metabolism of its major class member. Ulteriorly, the comparatively third dominant group in this study is Thermoplasmatota, which can encode copper membrane monooxygenases (CuMMOs) that mediate aerobic oxidation of ammonia, methane, and other hydrocarbons as substrates (Khadka et al. 2018), and is also important for the global carbon and nitrogen cycle (Diamond et al. 2022).

As for the rare group at phylum level, Altiarchaeota, Nanoarchaeota, and Hydrothermarchaeota belong to the DPANN superphylum. Altiarchaeota is adaptable to high‐temperature environments (Sabath et al. 2013), which is abundant in August samples. Nanoarchaeota and Hydrothermarchaeota prefer living in low oxygen environments, which may benefit from their adaptive strategies for nutrient‐limited niche, and they could exhibit rich genetic diversity when in hypoxic environments (Bird et al. 2016; Carr et al. 2019).

### 4.3 Driving Factors on Distribution of Archaeal Community

Due to the limitations of specific ecological niches, archaea communities are impacted by both abiotic and biotic factors (Cao, Auguet, and Gu 2013; Zheng et al. 2013). The exchanges exist between bottom water and sediment pore water under the movement of water, solutes, and particles. Using physic‐chemical parameters from both bottom water and pore water to analyze environmental influences on sediment microbial diversity is a well‐established method. The combination allows for a more comprehensive view of how water column conditions influence sediment ecosystems, aiding in the understanding of nutrient cycles, redox processes, and microbial community structures. As reported, long‐term warming alters microbial composition in surface sediments and modifies the geochemical profiles of overlying water, influencing nutrient cycling and microbial diversity at the sediment–water interface (Seidel et al. 2023; Broman et al. 2017). Additionally, nutrient concentration and redox gradients between bottom water and pore water create unique niches that support diverse microbial communities in reef sediments (Oh et al. 2022). These findings collectively underscore the importance of analyzing interactions between bottom water (measured using CTD instruments) and sediment pore water to understand their combined influence on sediment physicochemical properties. Consequently, physicochemical parameters from both bottom water and sediment pore water in the Bohai Sea hypoxic zone were integrated to investigate their impact on archaeal community diversity in this study.

Multiple environmental factors abiotically in Bohai sediments, such as DO, NO_3_^−^, NH_4_^+^, salinity (Figure 6), are key factors driving microbial community structure, although the seasonal trend of DO depletion is strongly driven by biological productivity. Archaea in turn responses differently in low oxygen zones. For example, the relative abundance of Thaumarchaeota, Euryarchaeota, and Thermoproteota increases in sediments of the northern Gulf of Mexico and the eastern South Pacific, where the DO concentration is < 2 μM (Belmar, Molina, and Ulloa 2011; Devereux et al. 2015; Lu et al. 2019); however, the relative abundance of Asgardarchaeota decreases with enhancement of hypoxia (Zou et al. 2022), although it dominates in the Yangtze River Estuary and the low DO site from this study. Furthermore, the effects of low DO on microbial communities can, to some extent, provide insights into the impact of sediment oxygen demand (SOD) and Redox potential (Eh) on these communities, as shifts in oxygen availability and redox conditions directly influence microbial composition and function. SOD refers to the rate at which sediments consume oxygen from the overlying water (Cheng et al. 2024). In hypoxic environments, SOD was elevated due to the decomposition of organic matter and the activity of microbial communities in the sediments, and an increase in SOD can further exacerbate oxygen depletion, as bottom sediments continuously consume oxygen (Cheng et al. 2024; Santoferrara et al. 2022); therefore, the anaerobic microorganisms dominated and consequently alters the composition of the microbial community. Eh is a key indicator for the oxidative or reductive state within water bodies or sediments (Lacroix et al. 2023; Peiffer et al. 2021; Lau et al. 2018). Lower Eh, indicating a more reduced environment, favors the growth of organisms capable of anaerobic metabolism in hypoxic zones (Lu and Imlay 2021; Dick and Meng 2023).

Archaea in sediment may be sensitive to nutrient quality and quantity supplied from the overlying water (Hoshino and Inagaki 2019). Ammonium serves as a primary nitrogen source for AOA, particularly from the phylum Thaumarchaeota, which thrive in sediments where NH₄^+^ concentrations are elevated. AOA participates in nitrification, converting NH₄^+^ to NO₂^−^, a critical step in the nitrogen cycle that regulates nitrogen availability in sediment ecosystems (Huang et al. 2021). Nitrite, an intermediate in both nitrification and denitrification, is also utilized in processes such as anaerobic ammonium oxidation (anammox), where it reacts with NH₄^+^ to form nitrogen gas (Wang et al. 2023). Although archaea are not directly involved in anammox, they contribute to the upstream supply of NO₂^−^ through ammonia oxidation. Under anoxic conditions, some archaeal species can also reduce NO₂^−^, thus playing a role in denitrification, which is crucial for nitrogen removal from sediments (Zhang, Ji, et al. 2023). NO_3_^−^ acts as a terminal electron acceptor for certain archaea in anoxic environments, supporting processes like NO₃^−^ reduction and denitrification. These processes allow archaeal communities to thrive where oxygen is scarce but NO_3_^−^ is available, contributing to nitrogen gas production and the regulation of nitrogen fluxes within the ecosystem (Martínez‐Espinosa 2020). Overall, the interplay between nitrogen compounds and archaeal activity drives essential nitrogen‐transforming processes within marine sediments, influencing redox conditions, nutrient availability, and microbial diversity. The balance between processes like nitrification and denitrification, often mediated by archaea, plays a crucial role in sediment biogeochemistry and affects broader ecological stability in marine environments (Wright and Lehtovirta‐Morley 2023; Zhang, Zha, et al. 2023). These recent studies highlight the ecological significance of nitrogen chemistry in shaping the structure and function of archaeal communities in sediments. Members of Thermoproteota encode *nir* (nitrite reductase) genes (Lazar et al. 2016), which may explain positive association identified in this study between NO_2_^−^ and them (Table S3), and suggesting the potential of archaea for DNRA to NH₄^+^ in low oxygen environments. Besides, Euryarchaeota and Hydrothermarchaeota are involved in active nitrogen cycling by encoding *nar* (nitrate reductase) genes in hypoxic sediment and mangrove (Zhou et al. 2019, 2020; Zhang et al. 2021), which may account for the positive correlation between NO_3_^−^ and Hydrothermarchaeota in this study (Table S3).

Salinity is another important factor structuring archaeal communities in this study, Asgardarchaeota and Halobacterota were associated with high salinity, which is in accordance with previous observations in hypoxic sediments such as Bay of Bengal, Pearl River Estuary, and Changjiang River Estuary (Wang et al. 2017; Zou et al. 2020, 2022). In addition, depth and temperature also shaped the archaeal community (Table S3). Variation of water depths in the Bohai Sea may lead to slight differences in the quality and quantity of organic matter in the sediments, resulting in staggered DO concentrations caused by different oxygen consumption (Lutz et al. 2007; Hoshino and Inagaki 2019).

Archaeal community is not only driven by multiple abiotic factors, but biotic essentials also contribute to the species' composition and distribution. Archaea exhibits complex dynamics with probable symbiotic by intertaxa interactions (Liu et al. 2020). In this study, as shown by networking analysis, within Thermoproteota, interactions between OTUs from various subgroups were extremely complicated, showing a high diversity and niche variations. Nitrososphaeria is mostly associated with other archaea, and it is capable of *amo* and *nir* genes transcription, transporters of nitroreductases, ureases, and urea, which may affect the nutritional conditions of their habitat environment, indicating a potentially significant impact on the archaeal community metabolize due to its clearly active application of nutrients (Vuillemin 2023). And Bathyarchaeia has the highest betweenness centrality in the interaction network (Table S4), which suggests their strong ability to control other archaea through metabolisms such as carbon cycles, acetogenesis, and fermentation, once again proving the competitiveness between Nitrososphaeria and Bathyarchaeia (Lazar et al. 2016; Zhou et al. 2018). Conversely, the relatively simple interactions among Halobacteriota may be consistent with their unitary methanogenic capacity use of b‐type cytochromes (Rinke et al. 2021).

On the other hand, correlations between archaea and bacteria also affect the diversity and distribution of archaeal communities. Thermoproteota is correlated with diverse bacteria including members of the Proteobacteria, Bacteroidetes, and Chlorobi phyla (Kuypers, Marchant, and Kartal 2018; Needham and Fuhrman 2016; Wang et al. 2019), which may account for their strong metabolic activity in various substrates such as aromatic compounds, methane, lignin, and extracellular carbohydrates (He et al. 2016; Meng et al. 2014; Qi et al. 2021). Asgardarchaeota is positively correlated with Acidobacterium and Proteobacterium as the second dominant group in this study (Figure 8B). This is in alignment with previous results found in South China Sea sediment (Zhang et al. 2021). The dynamics of microbial community composition and co‐occurrence patterns are not synchronized, and co‐occurrence patterns based on network properties cannot systematically and comprehensively reflect the correlations between real taxonomic units (Ma et al. 2016). Therefore, it is necessary to further determine the specific interactions between groups and link them to ecological functions.

### 4.4 The Function of Archaea in Bohai Sediment and its Impact on Nitrogen Cycling

Functional prediction provides possibility for deciphering the role of archaea in biogeochemical cycling in Bohai Sea. Since multiple metabolism pathways may cope with various environmental pressures (Mueller et al. 2021), the increasing trend of predicted pathways in August indicates the high environmental stress on archaea, which is the occurrence of low oxygen, revealed by MetaCyc database comparison (Figure 11). Higher metabolic activity in archaea communities under oxygen stress or limitation also had been reported in anoxic Namibian shelf sediments, Eastern Indian Ocean sediment, and hypoxic subterranean Vapor Cave (Orsi et al. 2020; Wang et al. 2017; Martin‐Pozas et al. 2020). It is corroborated by the composition of some archaea, such as Hydrothermarchaeota, Thaumarchaeota, and Nitrososphaeria (Thaumarchaeota/Thermoproteota), which were relatively abundant in August (Figure 4). Moreover, archaea in sediment are capable of mediating strong biosynthetic pathways, including the synthesis of nucleosides and nucleotides, amino acids, carbohydrates, and aromatic compounds, where related amino acids can regulate the growth and development of archaea at multiple growth stages (Tomita 2017). In addition, sedimentary archaea can also participate in product metabolism and energy generation, such as TCA cycle, fermentation, and electron transfer chains, and can also participate in the utilization and assimilation of C_1_ compounds. It makes significant contributions to archaea in response to environmental changes by synthesizing essential amino acids, including low oxygen (Wang et al. 2020). The findings above suggest a close association between organic matter and microbial communities. However, not all sedimentary organic matter originates from microbial sources. Microorganisms play a particularly crucial role in processing organic matter introduced from external sources.

Organic matter in marine sediments originates from multiple sources, including marine and terrestrial inputs. Marine‐derived organic matter, primarily from phytoplankton and other marine organisms, is rich in labile compounds such as lipids and proteins, making it easily degradable. In contrast, terrestrial inputs, including plant materials transported via rivers, consist of more recalcitrant compounds like cellulose and lignin, which are harder to break down (Avellan, Duarte, and Rocha‐Santos 2022; Szymczak‐Żyła and Lubecki 2022). Microbial communities play a crucial role in processing these inputs, since they not only decompose existing organic matter, but also produce metabolites that contribute to the organic matter pool (Oni et al. 2015; Sobek et al. 2023). These metabolites, such as fatty acids and alcohols, serve as substrates for other microbes, facilitating complex interactions and the cycling of elements like carbon and nitrogen (Johnson 2017). Recent studies underscore the adaptive nature of microbial consortia, which shift their metabolic pathways depending on the available substrates and environmental conditions, from aerobic degradation in surface sediments to anaerobic processes in deeper layers (Broman et al. 2022; Chen et al. 2023; Yin et al. 2024). This dynamic interplay between organic matter sources and microbial metabolism drives essential biogeochemical cycles within marine ecosystems.

Under hypoxic, nitrogen cycling is more sensitive than other elemental cycling (Fennel and Testa 2018), and archaea mainly not only mediate the step of oxidizing NH₄^+^ to NO_2_^−^ but also participate in nitrogen fixation and removal (Offre, Spang, and Schleper 2013). It is consistent with this study that nitrogen fixation function has been enhanced in July and continues to increase at Station A5, while decreasing at other stations. N_2_ fixation is a common characteristic of methanogenic archaea (Bräuer et al. 2020), and both *Methanobacterium* and Bathyarchaeia identified in this study belong to methanogenic archaea (Mei et al. 2022; Zheng et al. 2022), which are involved not only in nitrogen fixation but also sharing nitrogen with bacteria (Dekas, Poretsky, and Orphan 2009). In addition to the nitrogen fixation process, NO_2_^−^ accumulation has been measured in this study (Figure 5), indicating active denitrification in the hypoxic zone. Denitrifying microorganisms and NO_2_^−^ accumulation have been reported previously in the Arabian Sea and northern Chilean hypoxic waters, contributing to benthic nitrogen cycle (Stewart, Ulloa, and DeLong 2012; Lüke et al. 2016).

Furthermore, archaea play a crucial role in deep sea ecosystems, particularly in ocean sediments, by driving essential chemoautotrophic processes. These processes rely on the oxidation of inorganic molecules, allowing archaea to generate energy and fix carbon in the absent of sunlight. Such as *Thaumarchaeota*, the AOA, which transform NH₄^+^ into NO_2_^−^ and generate energy to support their chemoautotrophic metabolism, are contributing to global biogeochemical nitrogen and carbon cycling in great abundance and significance (Dekas et al. 2019; Gomez‐Saez et al. 2017; Wright and Lehtovirta‐Morley 2023; Zheng et al. 2024). Moreover, archaea also engage in methane metabolism in deep‐sea sediment. Methanogenic archaea, such as members of the Euryarchaeota and Thermoproteota (formerly Crenarchaeota), are often found in hydrothermal environments, which reduce carbon dioxide with hydrogen to produce methane, an important energy source for symbiotic organisms like tube worms and other benthic fauna (Gomez‐Saez et al. 2017). These chemoautotrophic processes ensure the recycling of key elements in marine sediments, maintaining ecosystem stability and supporting life in harsh, oxygen limited and even hypoxic environments (Qin et al. 2024). Advances in metagenomics, transcriptomics, and other techniques continue to uncover new insights into the ecological significance of these microorganisms (Pereira et al. 2024), showing that archaea are indispensable in sustaining deep‐sea life.

## 5 Conclusions

During oxygen depletion in the Bohai Sea, both environmental parameters and biological responses shifted, resulting in significant spatial and temporal changes in archaeal community structures from nonhypoxic to hypoxic sites. The dominant Thermoproteota (80.61%) decreased under low DO conditions, while Thermoplasmatota (5.27%) increased, particularly in nearshore areas. Asgardarchaeota (8.70%), especially Lokiarchaeia, declined in hypoxic sites, whereas rare groups such as Halobacteriota and Methanobacteriota showed significant increase tendency as hypoxia intensified. DO and NO₃^−^ emerged as primary environmental drivers in these communities. Functionally, archaea in Bohai Sea sediments adapted to hypoxia by prioritizing metabolic and genetic information processes, with a proportion of over 90% among predicted functions. Key pathways including nitrogen cycling processes, such as ammonia oxidation, DNRA, and nitrification, varied along a nearshore–offshore gradient and indicated adaptive responses to low‐oxygen conditions. These findings underscore the critical role of archaea in maintaining nitrogen and carbon cycles under hypoxia, with pathways like aerobic respiration, amino acid biosynthesis, and an incomplete TCA cycle that supports ecosystem resilience.

## Author Contributions

**Xiaoxiao Guo:** data curation (equal), formal analysis (equal), software (equal), visualization (supporting), writing – original draft (lead). **Yanying Li:** data curation (equal), formal analysis (equal), software (equal), visualization (lead), writing – original draft (supporting). **Guisheng Song:** resources (lead), writing – review and editing (supporting). **Liang Zhao:** resources (lead). **Jing Wang:** conceptualization (lead), funding acquisition (lead), resources (equal), supervision (lead), writing – review and editing (lead).

## Ethics Statement

The authors have nothing to report.

## Consent

The authors have nothing to report.

## Conflicts of Interest

The authors declare no conflicts of interest.

## Supporting information


**Figure S1.** Rarefaction curve of 16S rRNA from archaea in sediment.
**Figure S2.** Hierarchical cluster analysis of samples in Bohai Sea.
**Figure S3.** Correlation analysis between archaea alpha diversity index and DO.
**Figure S4.** Correlation analysis between archaea beta diversity index and DO.
**Table S1.** Sample information collected from hypoxic zones in the Bohai Sea.
**Table S2.** VIF of sedimentary physicochemical parameters.
**Table S3.** Spearman correlation analysis between physicochemical parameters and abundance of 16S rRNA genes, alpha diversity index, respectively.
**Table S4.** Topological properties of archaeal networks.

## Data Availability

The raw sequencing data of 16S rDNA genes were deposited in the SRA database at NCBI under accession nos.: SRR28720242–SRR28720256 (PRJNA1100295) https://www.ncbi.nlm.nih.gov/bioproject/PRJNA1100295.
