# Supplementary material for: Adaptation of Archaeal Communities to Summer Hypoxia in the Sediment of Bohai Sea
Source: Ecol Evol. 2025 Jan 8;15(1):e70768. doi: 10.1002/ece3.70768 (PMC11707553; doi:10.1002/ece3.70768)
Supplement: Supplementary file 1 — Figure S1. Rarefaction curve of 16S rRNA from archaea in sediment. Figure S2. Hierarchical cluster analysis of samples in Bohai Sea. Figure S3. Correlation analysis between archaea alpha diversity index and DO. Figure S4. Correlation analysis between archaea beta diversity index and DO. Table S1. Sample information collected from hypoxic zones in the Bohai Sea. Table S2. VIF of sedimentary physicochemical parameters. Table S3. Spearman correlation analysis between physicochemical parameters and abundance of 16S rRNA genes, alpha diversity index, respectively. Table S4. Topological properties of archaeal networks. [file ECE3-15-e70768-s001.pdf]

Supplementary materials

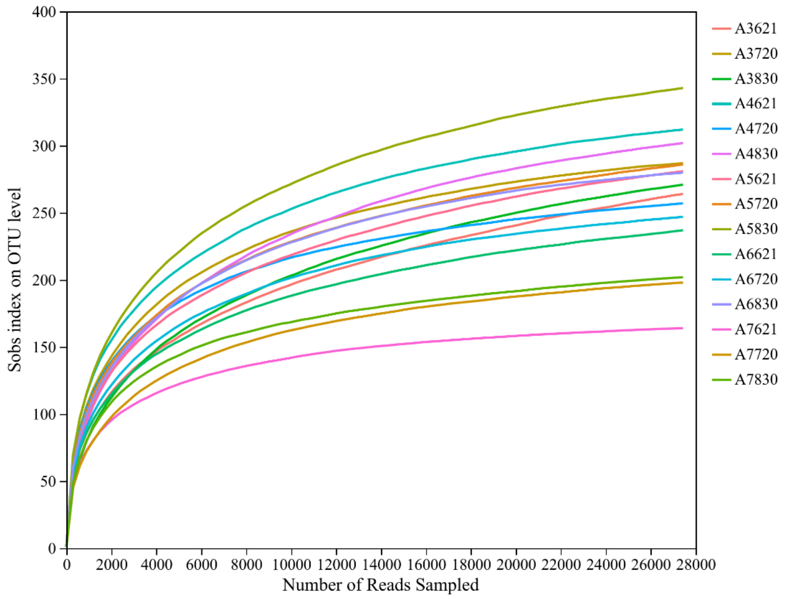

FIGURE S1 Rarefaction curve of 16S rRNA from archaea in sediment.

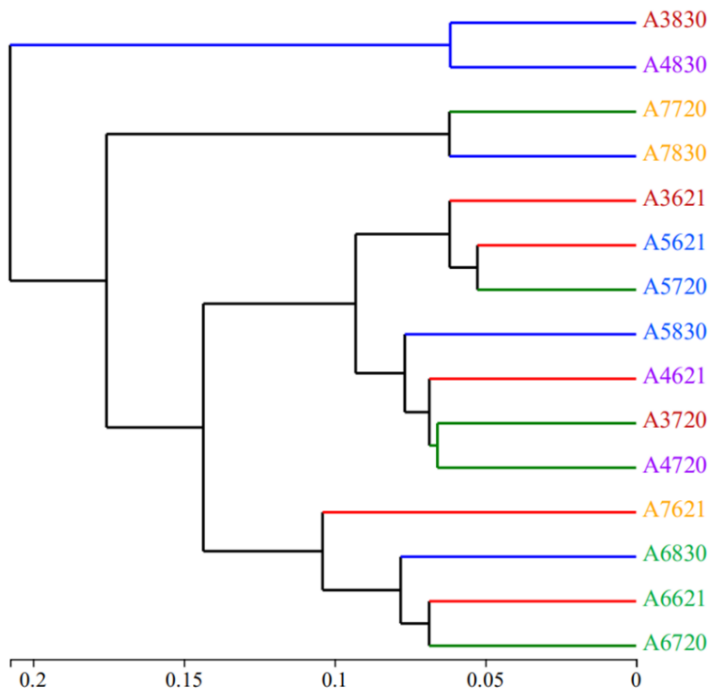

FIGURE S2 Hierarchical cluster analysis of samples in Bohai Sea.

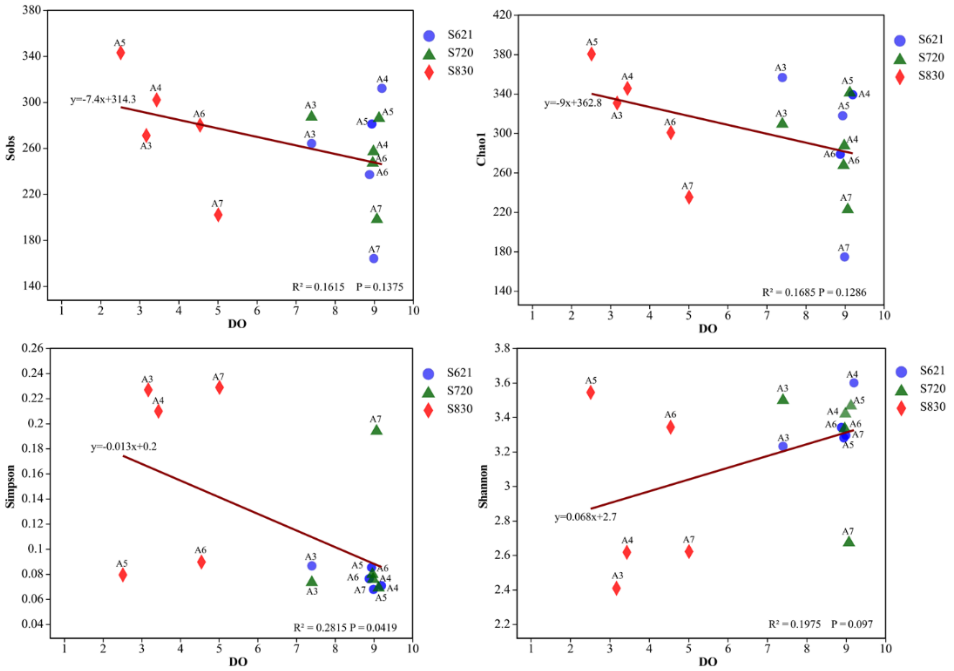

FIGURE S3 Correlation analysis between archaea alpha diversity index and DO.

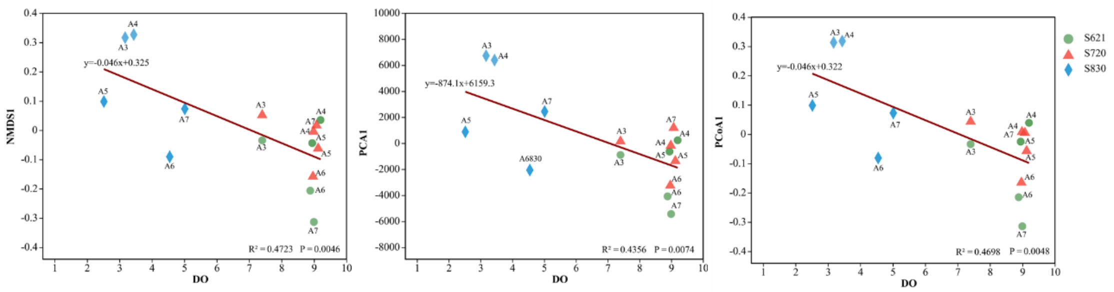

FIGURE S4 Correlation analysis between archaea beta diversity index and DO.

TABLE S1 Sample information collected from hypoxic zones in the Bohai Sea

Time	Sites	Location	Sequence number	Depth(m)	Temperature(°C)	Salinity	DO(mg/L)	pH	NO_2^- (mg/kg)	NO_3^- (mg/kg)	NH_4^+ (mg/kg)
21, June, 2021	A3	119.840E 39.6183N	54278	18.16	13.26	32.03	7.40	7.98	0.21	1.04	5.02
	A4	120.046E 39.5483N	54328	21.23	11.34	32.12	9.20	8.19	0.30	0.86	4.83
	A5	120.252E 39.4783N	45364	22.90	11.50	32.43	8.95	7.85	0.28	1.41	13.49
	A6	120.458E 39.4083N	45578	16.61	13.84	31.91	8.89	7.91	0.27	0.78	4.31
	A7	120.551E 39.3765N	49524	19.43	15.86	31.80	9.00	7.96	0.22	0.68	14.28
20, July, 2021	A3	119.840E 39.6183N	48089	14.96	17.52	31.63	7.40	7.94	0.89	2.49	0.12
	A4	120.046E 39.5483N	41629	20.92	15.57	31.64	8.99	7.89	0.89	2.50	0.12
	A5	120.252E 39.4783N	49442	23.50	13.25	31.94	9.13	7.85	0.89	2.50	0.12
	A6	120.458E 39.4083N	50204	18.06	15.67	31.77	8.97	7.73	0.56	1.56	0.07
	A7	120.551E 39.3765N	49941	17.90	17.56	31.75	9.08	7.76	0.49	1.38	0.06
30, August, 2021	A3	119.840E 39.6183N	43756	16.97	20.77	31.31	3.18	7.84	2.58	17.33	17.47
	A4	120.046E 39.5483N	43283	20.35	19.12	31.64	3.44	7.81	2.39	6.42	6.26
	A5	120.252E 39.4783N	51626	22.88	19.26	31.34	2.52	7.75	2.36	3.87	7.21
	A6	120.458E 39.4083N	44617	17.43	23.68	30.84	4.55	8.07	2.36	6.67	7.04
	A7	120.551E 39.3765N	46579	19.54	24.00	30.86	5.02	8.12	2.57	1.75	7.19
significance test				*p*	*p*	*p*	*p*	*p*	*p*	*p*	*p*
			season	0.94	0.00	0.00	0.00	0.26	0.00	0.03	0.00
			station	0.00	0.71	0.76	0.96	0.77	1.00	0.63	0.89

TABLE S2 VIF of sedimentary physicochemical parameters

Physical and chemical parameters	Depth	Salinity	DO	pH	NO_3^-	NH_4^+
VIF value	1.28	2.84	3.59	1.33	2.60	1.76

$VIF_i=1/(1-R_i^2)$. R_i is the Multiple Correlation Coefficient of the independent variable x_i for regression analysis of the other independent variables.

TABLE S3 Spearman correlation analysis between physicochemical parameters and abundance of 16S rRNA genes, alpha diversity index, respectively.

	Depth	DO	NH_4^+	NO_2^-	NO_3^-	pH	Salinity	Temperature
16S rRNA gene abundance								
Archaea	-0.03	0.32	-0.34	-0.13	-0.30	0.12	0.00	0.10
Diversity indices								
Chao	0.40	-0.36	0.19	0.18	0.42	-0.11	0.13	-0.19
Heip	0.01	0.54*	-0.29	-0.54*	-0.51	0.26	0.35	-0.48
Shannon	0.24	0.32	-0.32	-0.20	-0.09	0.19	0.18	-0.39
Shannoneven	0.14	0.57*	-0.24	-0.48	-0.44	0.31	0.36	-0.50
Simpson	-0.25	-0.61*	0.24	0.51	0.42	-0.18	-0.50	0.63*
Sobs	0.39	-0.27	0.09	0.29	0.47	-0.08	0.01	-0.14
Relative abundance of total archaeal phyla								
Aenigmarchaeota	-0.09	-0.25	-0.09	0.28	0.14	0.11	-0.46	0.41
Altiarchaeota	-0.18	-0.27	-0.01	0.18	0.06	-0.08	-0.38	0.43
Asgardarchaeota	0.13	0.64*	-0.28	-0.57*	-0.48	-0.08	0.52*	-0.52*
Crenarchaeota	-0.38	-0.23	0.26	0.15	-0.06	0.07	-0.23	0.42
Euryarchaeota	0.09	-0.38	-0.06	0.47	0.60*	-0.38	-0.24	0.09
Hadarchaeota	0.06	-0.19	0.19	0.37	0.00	0.37	-0.37	0.43

Halobacterota	0.14	0.38	-0.41	-0.43	-0.37	0.08	0.55*	-0.65*
Hydrothermarchaeota	0.27	-0.31	0.07	0.38	0.55*	-0.12	-0.13	-0.05
Iainarchaeota	0.47	-0.15	-0.14	0.20	0.48	-0.26	0.06	-0.25
Micrarchaeota	0.10	-0.48	0.07	0.48	0.70*	-0.23	-0.32	0.14
Nanoarchaeota	0.15	-0.39	0.22	0.33	0.55**	-0.15	-0.06	-0.03
Thermoplasmatota	0.31	-0.23	-0.06	0.25	0.43	-0.08	-0.02	-0.17
Unclassified	0.31	0.18	-0.05	-0.41	-0.20	0.19	0.42	-0.49

*Significantly correlated at 0.05 level. ** Significantly correlated at 0.01 level.

TABLE S4 Topological properties of archaeal networks

Kingdom	Phylum	Class	node id	degree	weight degree	closeness centrality	betweenness centrality	eigenvector centrality
Archaea	Crenarchaeota	Bathyarchaeia	OTU1	21	16.25	0.003	90.75	0.55
Archaea	Crenarchaeota	Bathyarchaeia	OTU120	18	13.28	0.003	101.50	0.42
Archaea	Crenarchaeota	Bathyarchaeia	OTU126	1	0.66	0.003	0.00	0.00
Archaea	NA	NA	OTU138	9	6.40	0.003	85.00	0.06
Archaea	Crenarchaeota	Nitrososphaeria	OTU150	31	23.82	0.003	104.50	1.00
Archaea	Crenarchaeota	Nitrososphaeria	OTU181	22	16.66	0.003	110.00	0.78
Archaea	Crenarchaeota	Nitrososphaeria	OTU200	13	9.38	0.003	127.33	0.10
Archaea	Crenarchaeota	Nitrososphaeria	OTU227	0	0.00	0.000	0.00	0.01
Archaea	Thermoplasmatota	Thermoplasmata	OTU229	8	6.12	0.003	8.00	0.16
Archaea	Crenarchaeota	Bathyarchaeia	OTU232	17	14.01	0.003	2.00	0.57
Archaea	Crenarchaeota	Bathyarchaeia	OTU233	9	6.59	0.003	49.25	0.12
Archaea	Thermoplasmatota	Thermoplasmata	OTU234	20	15.83	0.003	25.00	0.72
Archaea	Asgardarchaeota	Odinarchaeia	OTU236	8	5.92	0.003	4.25	0.27
Archaea	Thermoplasmatota	NA	OTU238	17	12.70	0.003	44.00	0.60
Archaea	Crenarchaeota	Bathyarchaeia	OTU239	3	2.15	0.003	0.00	0.04
Archaea	Crenarchaeota	Bathyarchaeia	OTU243	26	19.20	0.003	178.00	0.83
Archaea	Crenarchaeota	Nitrososphaeria	OTU246	11	7.52	0.003	87.00	0.30
Archaea	Thermoplasmatota	Thermoplasmata	OTU248	16	12.37	0.003	34.50	0.58
Archaea	Crenarchaeota	Bathyarchaeia	OTU252	7	5.23	0.003	19.00	0.04
Archaea	Crenarchaeota	Bathyarchaeia	OTU266	10	6.91	0.003	57.67	0.22
Archaea	Asgardarchaeota	Heimdallarchaeia	OTU268	16	11.90	0.003	15.00	0.58
Archaea	Thermoplasmatota	Thermoplasmata	OTU273	14	11.00	0.003	4.00	0.44
Archaea	NA	NA	OTU274	3	2.04	0.003	24.00	0.06
Archaea	Thermoplasmatota	Thermoplasmata	OTU275	18	13.86	0.003	32.00	0.63
Archaea	Crenarchaeota	Bathyarchaeia	OTU277	8	5.87	0.003	14.25	0.04
Archaea	Hydrothermarchaeota	Hydrothermarchaeia	OTU280	22	17.36	0.003	118.00	0.80
Archaea	Crenarchaeota	Nitrososphaeria	OTU286	12	9.34	0.003	23.00	0.49

Archaea	Crenarchaeota	Bathyarchaeia	OTU287	5	3.35	0.003	80.00	0.02
Archaea	Thermoplasmatota	Thermoplasmata	OTU293	8	6.21	0.003	0.00	0.31
Archaea	Crenarchaeota	Bathyarchaeia	OTU299	0	0.00	0.000	0.00	0.01
Archaea	Crenarchaeota	Bathyarchaeia	OTU302	1	0.68	0.003	0.00	0.01
Archaea	Crenarchaeota	Bathyarchaeia	OTU309	15	10.73	0.003	61.08	0.44
Archaea	Crenarchaeota	Bathyarchaeia	OTU33	8	5.64	0.003	40.00	0.10
Archaea	Thermoplasmatota	Thermoplasmata	OTU339	9	7.19	0.003	6.00	0.24
Archaea	Crenarchaeota	Bathyarchaeia	OTU35	8	5.67	0.003	48.00	0.13
Archaea	Crenarchaeota	Bathyarchaeia	OTU352	14	10.46	0.003	75.00	0.15
Archaea	Crenarchaeota	Bathyarchaeia	OTU363	6	4.49	0.003	20.00	0.08
Archaea	Asgardarchaeota	Lokiarchaeia	OTU370	10	7.51	0.003	10.00	0.17
Archaea	Crenarchaeota	Bathyarchaeia	OTU38	16	12.33	0.003	28.00	0.50
Archaea	Crenarchaeota	Bathyarchaeia	OTU4	18	14.32	0.003	63.00	0.54
Archaea	Crenarchaeota	Nitrososphaeria	OTU402	6	4.18	0.003	66.00	0.19
Archaea	Crenarchaeota	Bathyarchaeia	OTU413	15	11.29	0.003	58.00	0.21
Archaea	Crenarchaeota	Bathyarchaeia	OTU415	12	8.49	0.003	186.50	0.09
Archaea	Thermoplasmatota	Thermoplasmata	OTU420	17	13.17	0.003	18.00	0.57
Archaea	Crenarchaeota	Bathyarchaeia	OTU428	9	6.63	0.003	54.67	0.14
Archaea	NA	NA	OTU433	1	0.66	0.003	0.00	0.00
Archaea	Asgardarchaeota	Lokiarchaeia	OTU443	15	11.57	0.003	47.00	0.50
Archaea	Crenarchaeota	Bathyarchaeia	OTU45	4	2.72	0.003	75.00	0.02
Archaea	Thermoplasmatota	Thermoplasmata	OTU455	20	16.16	0.003	28.00	0.73
Archaea	Thermoplasmatota	Thermoplasmata	OTU473	19	15.10	0.003	52.00	0.66
Archaea	Crenarchaeota	Nitrososphaeria	OTU481	18	13.53	0.003	25.50	0.66
Archaea	Crenarchaeota	Bathyarchaeia	OTU506	19	14.51	0.003	80.50	0.48
Archaea	NA	NA	OTU516	6	4.23	0.003	19.00	0.13
Archaea	Crenarchaeota	Bathyarchaeia	OTU517	20	15.22	0.003	78.00	0.55
Archaea	Crenarchaeota	Bathyarchaeia	OTU522	18	13.77	0.003	141.50	0.50
Archaea	Crenarchaeota	Bathyarchaeia	OTU526	11	8.26	0.003	30.00	0.28
Archaea	Halobacterota	Methanosarcinia	OTU53	2	1.46	0.003	0.00	0.01
Archaea	Asgardarchaeota	Lokiarchaeia	OTU534	18	14.39	0.003	41.25	0.61
Archaea	Asgardarchaeota	Odinarchaeia	OTU539	12	8.69	0.003	12.00	0.30
Archaea	NA	NA	OTU550	10	7.29	0.003	58.00	0.21
Archaea	Crenarchaeota	Bathyarchaeia	OTU556	0	0.00	0.000	0.00	0.01
Archaea	Asgardarchaeota	Lokiarchaeia	OTU566	15	10.97	0.003	67.42	0.46
Archaea	NA	NA	OTU582	3	2.11	0.003	25.00	0.02
Archaea	Crenarchaeota	Bathyarchaeia	OTU585	12	8.80	0.003	67.00	0.06
Archaea	Crenarchaeota	Bathyarchaeia	OTU6	14	10.06	0.003	71.58	0.30
Archaea	NA	NA	OTU69	11	8.14	0.003	34.00	0.33
Archaea	Crenarchaeota	Bathyarchaeia	OTU77	2	1.36	0.003	0.00	0.05
Archaea	Crenarchaeota	Bathyarchaeia	OTU81	18	13.47	0.003	49.00	0.64
Archaea	Crenarchaeota	Bathyarchaeia	OTU86	2	1.29	0.003	1.00	0.04
Archaea	Crenarchaeota	Bathyarchaeia	OTU87	4	2.75	0.003	1.50	0.01
Archaea	Crenarchaeota	Bathyarchaeia	OTU96	19	15.01	0.003	22.50	0.46
